# The PACT advance decision-making template: preparing for Mental Health Act reforms with co-production, focus groups and consultation

**DOI:** 10.1016/j.ijlp.2020.101563

**Published:** 2020

**Authors:** Lucy A. Stephenson, Tania Gergel, Alex Ruck Keene, Larry Rifkin, Gareth Owen

**Affiliations:** a Institute of Psychiatry, Psychology and Neuroscience, King's College London, United Kingdom; b Institute of Psychiatry, Psychology and Neuroscience, King's College London, 39 Essex Chambers, United Kingdom; c South London and Maudsley NHS Foundation Trust, United Kingdom

**Keywords:** Advance directive, Severe mental illness, Bipolar, Mental capacity, Mental health law

## Abstract

**Background:**

Advance decision making (ADM) in mental health is supported by stakeholders but faces significant barriers. These must be overcome, not least to support the UK government's commitment to introduce statutory mental health ADM in England and Wales.

**Aims:**

To build understanding and address the gap between aspirations for ADM and actuality, with feasible co-produced ADM resources.

**Methods:**

We used focus groups and consultation to explore experience and views of stakeholders on ADM processes and materials. Discussions included feedback on an ADM template which was adapted accordingly throughout the research process.

**Results:**

Between September 2017 and December 2019, 94 individuals, representing stakeholders advised on design and process of ADM, alongside wider discussion at stakeholder events. Collaborative ADM was universally supported. Valued outcomes were diverse and combining aspirations with practicality required resolving dilemmas. A prototype template and guidance, the PACT (Preferences and Advance decisions for Crisis and Treatment) was co-produced, designed to help manage fluctuating mental capacity through collaborative decision making. The PACT enables direct engagement with medico-legal frameworks, with provision to facilitate person-centred assessments, treatment refusals and requests. Resources including supported engagement and cross-agency awareness and accessibility were seen as essential.

**Conclusion:**

Our research confirms high stakeholder motivation to engage in ADM is hampered by multiple barriers. We identified enabling conditions for ADM and co-produced an ADM template and guidance which supports achievement of a range of valued outcomes. Further developments to support and evaluate the process of implementation are now needed to prepare for statutory change.

## Highlights

• Mental health advance decision-making is widely approved but difficult to implement.
• UK government has committed to legal reform supporting advance decision-making.
• To prepare, stakeholder concerns need to be understood and addressed.
• Stakeholders were invited to co-produce the PACT advance decision-making template.
• PACTs facilitate collaborative, personalised advance decision making.

## 1 Introduction

Advance decision making (ADM) for mental health crises is widely approved, but under-used and under-resourced, particularly when compared to ADM in physical healthcare settings. Existing research on ADM in severe mental illness (SMI) has confirmed interest amongst key stakeholders (Hindley et al., 2019; Swanson, Swartz, Ferron, Elbogen, & van Dorn, 2006b), together with some effectiveness in reducing coercion and increasing therapeutic alliance (de Jong et al., 2016; Molyneaux et al., 2019; Thornicroft et al., 2013). However, difficulties in achieving uptake, engagement with legal provision and successful implementation are consistent (Hindley et al., 2019; Morriss, Mudigonda, Bartlett, Chopra, & Jones, 2017; Swanson et al., 2006b) and there is limited exploration of the diversity of potential outcomes beyond a reduction in compulsory admission. This situation is surprising given the relapsing/remitting pattern of many severe mental illnesses (SMI) lends itself to the development of highly informed ADM which harness service user lived expertise of repeated mental health crises.

### 1.1 Reform of the Mental Health Act 1983 to include statutory provision for Advance Decision Making

In England and Wales government has recently committed to introducing statutory provision for ADM using ‘Advance Choice Documents’ (ACDs) in upcoming Mental Health Act 1983 (MHA) reform. Unlike existing ADM provision within the Mental Capacity Act 2005 (MCA), ACDs will retain legal weight even for those detained under the MHA. This important, and service user supported (Rethink Mental Illness, 2017), policy aims to increase parity between the treatment of mental and physical illness (Owen et al., 2019).

### 1.2 Statutory provision for mental health Advance Decision Making supports human rights

Achieving legal parity for people with any form of disability is a human rights issue. Article 12 of the United Nations Convention on the Rights of Persons with Disabilities (UNCRPD) states that ‘persons with disabilities enjoy legal capacity on an equal basis with others in all aspects of life’ (United Nations General Assembly, 2007). Therefore, introducing provision for formal mental health ADM that mirrors physical health ADM can be understood as redressing the balance and moving closer to the standards set in the UNCRPD.

However, this perspective has some complications, given that this proposed legal reform is seen as a way of increasing autonomy through the ethical concept of ‘precedent autonomy’. This allows individuals to extend autonomy to future periods when their capacity for informed decision-making is impaired, through ADM when they are still well/ retain capacity. Here, ADM documents made by the service user when they had DMC-T would be activated when they lose DMC-T due to an episode of mental illness. This version of ‘precedent autonomy’ depends on accepting that an individual could be assessed as having or not having mental capacity due to disability, an idea itself viewed as a violation of the fundamental human rights for autonomy by the United Nations Committee on the Rights of Persons with Disabilities.

The Committee's General Comment 1 on Article 12 of the UNCRPD states that ‘perceived or actual deficits in mental capacity must not be used as justification for denying legal capacity’ and calls for the abolition of ‘substitute decision-making’ within mental health laws. A full discussion of the Committee's interpretation of the UNCRPD and whether using the notion of mental capacity advances or diminishes the aims of the articles of the UNCRPD is beyond the scope of this article.^2^ However, a key point in the debate for mental health ADM is around whether ADM can be understood as support for the exercise of legal capacity and autonomy for purposes of Article 12, given that it operates across time rather than being the expression of that legal capacity at the specific point where the decision may be required.

This may be a particularly contentious issue in the context of ‘self-binding’ advance decisions where a person makes an advance request for coercive treatment in acknowledgement that at the time when the treatment is required i.e. during an episode of illness they are likely to refuse it (Das et al., 2020).

Whatever the position on the notion of mental capacity, ADM is widely understood as a tool which can help service users realise the ideals of Article 12 but one that is challenging to implement, a difficulty recognised across multiple jurisdictions (Ward, 2017). Given the known challenges in implementing mental health ADM our research takes a pragmatic approach and aims to understand, anticipate and address the challenges within the current and reformed legal framework to enable service users to realise the benefit of these new legal provisions and support their right to exercise their legal capacity on an equal basis with others.

### 1.3 Study rationale and objectives

For a broad exploration of the complex subjective phenomena that may influence stakeholders' motivation and experience of engaging with ADM, we conducted a focus group study. This aimed to deepen understanding of desired outcomes, enabling conditions and barriers, while also developing co-produced ADM materials, which maximise engagement with current law (in England and Wales) and can be easily adapted to work as ACDs or within other medico-legal frameworks. Materials were further refined during a consultation process. We have already made these resources available for use in local QI and research projects, to aid future phases of outcome and process evaluation and implementation. We now aim to make them more widely available, in response to increasing stakeholder demand from both individuals and organisations.

## 2 Material and methods

### 2.1 Study design and participants

Based on the view that understanding ADM in a ‘paradigm’ case of fluctuating capacity (Owen et al., 2011) can create processes to support ADM in a broader and more complex range of contexts, we focussed on bipolar. The multidisciplinary research team included expertise in psychiatry, medical ethics, law, psychotherapy and lived experience of ADM in bipolar. Public and patient involvement (co-production) has been fundamental at all stages: within the core research team and through ongoing consultation with a Service User Advisory Group and third sector organisations.

The research team internally co-produced a prototype ADM template, using a conceptual model of collaborative ADM (Gergel & Owen, 2015) and informed by a survey of over 900 members of the leading national service user charity for people with bipolar (Bipolar UK) (Hindley et al., 2019) and a literature review. This prototype was presented to legal experts to ensure compatibility with existing legal frameworks.

Focus groups, a method considered useful for eliciting views from hard to reach groups, safe discussion of difficult topics, problem solving and generating new ideas (Barbour, 2008) were then held. The aim was both to explore participants' experience and opinions on ADM and to help develop the ADM materials. Participants were purposively selected from key stakeholder groups: service users (SU) with bipolar, family members/friends of service users with bipolar (F&F), consultant psychiatrists (P), AMHPs (S), and care coordinators (C). SU and F&F participants were recruited from Bipolar UK and from participants in previous studies who had signalled interest in future research participation. Clinicians were recruited by email contact with individuals from South London and Maudsley NHS Foundation Trust and professional training organisation lists. We held 7 focus groups with 10 service user, 3 family member and 19 clinician participants. Written informed consent was obtained from all subjects.

The authors assert that all procedures contributing to this work comply with the ethical standards of the relevant national and institutional committees on human experimentation and with the Helsinki Declaration of 1975, as revised in 2008. All procedures involving human subjects/patients were approved by London – Surrey borders research ethics committee (ref: 17/LO/1071).

Following analysis of focus group data, a second prototype template, draft care pathway and clinician and service user guidance documents were co-produced and further refined through a consultation process involving 5 service user led organisations and 5 frontline community mental health teams at South London and Maudsley NHS Foundation Trust. Overall, 94 individuals took part in the focus groups and consultation process, as well as presentations at national clinical and service user conferences.

For further details on participants see Table 1a, Table 1b, Table 1c, Table 1d, Table 1e, Table 1f, Table 1g.

**Table 1a:** Characteristics of service user focus groups.

Service Users focus groups n=10		n
Age	Mean years (s.d.)	42.1 years (12.3)
Gender	Male	3
	Female	7
Ethnicity	White British	4
	Other White	1
	Black British/Caribbean/other/mixed	0
	Asian/mixed Asian	2
	Other	1
	Prefer not to say	2
Relationship status	In a relationship	2
	Not in a relationship	7
	Prefer not to say	1
Education	Diploma	1
	Undergraduate qualification	4
	Postgraduate qualification	4
	Prefer not to say	1
Employment	Employed	3
	Unemployed	2
	Long term sickness	3
	Prefer not to say	2
Benefits	Receives benefits	6
	Does not receive benefits	2
	Prefer not to say	2
Diagnosis	Bipolar 1	5
	Bipolar 2	3
	Cyclothymia	1
	Unsure	1
Hospitalisation	Never been hospitalised	6
	Several hospitalisations	4
Detentions under MHA	Never been detained	7
	Several detentions	3
Current service use (may specify more than 1 service)	Primary care only	3
	Community Mental Health Team	5
	Specialist service	3
	Private care	2
	Third sector	2

**Table 1b:** Characteristics of friends and family focus group.

Friends and Family focus group n=3		n
Age	Mean years (s.d.)	42.7 years (17.8)
Gender	Male	2
	Female	1
Ethnicity	White British	2
	Other White	0
	Black British/Caribbean/other/mixed	1
	Asian/mixed Asian	0
Relationship status	In a relationship	2
	Not in a relationship	1
Education	Undergraduate qualification	2
	Postgraduate qualification	1
Employment	Employed	1
	Retired	1
	Long term sickness	1
Benefits	Receives benefits	1
	Does not receive benefits	2
Loved one’s diagnosis	Bipolar 1	2
	Bipolar 2	1
Nature of relationship	Partner	1
	Parent	1
	Child	1
Hospitalisation	Never been hospitalised	0
	Several hospitalisations	3
Detention under MHA	Never been detained	0
	Several detentions	3

**Table 1c:** Characteristics of clinician focus group.

Clinician focus groups n=19		n
Gender	Female	8
	Male	11
Ethnicity	White British	12
	Other White	3
	Black British/Caribbean/other/mixed	1
	Asian/mixed Asian	2
	Other	1
	Prefer not to say	0
Clinical setting	CMHT	8
	Specialist affective disorder service	1
	Specialist perinatal service	1
	Liaison service	1
	Primary care	1
	Crisis service	3
	Inpatient	2
	Social services	1
	Other	1
Role	Consultant Psychiatrist	7
	Care Coordinator	6
	AMHP	6

**Table 1d:** Characteristics of legal experts.

Consultation with legal experts n=3		n
Gender	Female	1
	Male	2
Role	Solicitor	1
	Barrister	2
Specialism	Mental health law	3
	Mental capacity law	3

**Table 1e:** Consultation with service user led organisations.

Consultation with service user led organisations n=5		
Organisation	Description	No of individuals consulted
McPin Foundation (UK)	Conducts user focused mental health research and builds the capacity of others to conduct user focused research	3
Mental Health and Justice Project Service User Advisory Group (London)	A group of 10 people with lived experience of a range of mental health conditions who meet regularly to advise on research across the Mental Health and Justice Project. This groups is hosted and led by the McPin Foundation.	10
Bipolar UK (UK)	National UK charity dedicated to supporting people with bipolar with a focus on peer support.	3
South London and Maudsley (SLAM) Recovery College (South East London)	Offers workshops and courses to SLAM service users and staff that are co-designed and co-run by trainers with lived experience and professional experience.	2
Promise Resource Network (USA)	Extensive expertise and experience with providing peer support to service users who wish to create Psychiatric Advance Directives within existing US legal frameworks	2

**Table 1f:** Consultation with additional interested individual stakeholders.

Consultation with additional interested individual stakeholders n=10	n
Service users	4
Friends/family members	4
Health professionals	2

**Table 1g:** Consultation with frontline multidisciplinary clinical teams.

Consultation with frontline multidisciplinary clinical teams (South east London) n=5			
Type of team	Number of teams	Description	Number of individuals consulted
Psychosis focussed community mental health teams	3	Multidisciplinary mental health community teams including psychiatrists, mental health nurses, social workers, psychologists and occupational therapists working with people who have long term severe mental illnesses such as bipolar and schizophrenia	18
Specialist affective disorder team	1	Multidisciplinary mental health community team providing specialist care to people with bipolar	5
Perinatal specialist team	1	Multidisciplinary mental health community team providing care to women with severe mental illnesses who are pregnant and in the first year after birth	6

### 2.2 Data collection

#### 2.2.1 Focus groups

Focus groups, held between September 2017 and November 2018, were co-facilitated by LS and TG, with 2 also observed by GO. The facilitators' broad range of professional and lived experience and expertise allowed the experience and views of diverse participants to be clearly understood and deeply probed, within a secure and open environment. To provide as neutral an environment as possible, groups were held in university facilities. All groups started with a video explaining ADM and a chance to request clarification of key concepts. Discussion of previous experience and opinions on ADM used a topic guide, developed by the research team and tailored to the nature of the participant group (Krueger & Casey, 2002). Participants were then shown the template and encouraged to provide candid feedback.

#### 2.2.2 Consultation process

Structured field notes were taken during and immediately following consultation sessions conducted by LS and TG. Materials were also distributed to key stakeholders and any written or verbal feedback recorded.

### 2.3 Analysis

#### 2.3.1 Focus groups

Focus groups were audio recorded, transcribed and entered into coding software (NVivo 12). We used thematic analysis, (Braun & Clarke, 2006) to achieve flexibility to identify themes across a diverse dataset. The ‘trustworthiness’ of analysis was checked using criteria developed by Nowell et al. (Nowell, Norris, White, & Moules, 2017) and SRQR reporting guidelines were followed (O'Brien, Harris, Beckman, Reed, & Cook, 2014).

LS and TG read the raw data independently, discussed initial reflections then developed a preliminary coding framework. An inductive approach was used and both coding framework and themes were refined through an iterative process until saturation was reached. A thematic map was then used to create a logic model. This was refined by the research team until consensus was reached that themes and model accurately represented the data. Updated drafts of the template, accommodating feedback, were presented to successive groups, and revisions made until all team members were satisfied with the prototype template and supporting materials. Ongoing research was discussed and reflected upon at frequent team meetings throughout this process.

#### 2.3.2 Consultation process

Feedback from the consultation process was discussed until team members agreed that all feedback had been captured and appropriately translated into development of the ADM model and materials.

### 2.4 Role of the funding source

The funder of this study had no role in the collection, analysis, or interpretation of data and no role in writing the report or submitting for publication. The corresponding author had full access to study data and final responsibility for decisions surrounding publication.

## 3 Results

Participants were united by enthusiasm for mental health ADM despite diverse motivations and aspirations. One predominant concern was how to reconcile aspirations with resource constraints and ensure clinical, legal and social practicability, in order to avoid unrealistic expectations about the accessibility and implementation of documents. From the initial legal consultation, the PACT was seen as a document to ‘inform, not fetter, clinical judgement’, and ways to avoid concerns about restricting exercise of clinical judgement were explored throughout.

**Fig. 1:**
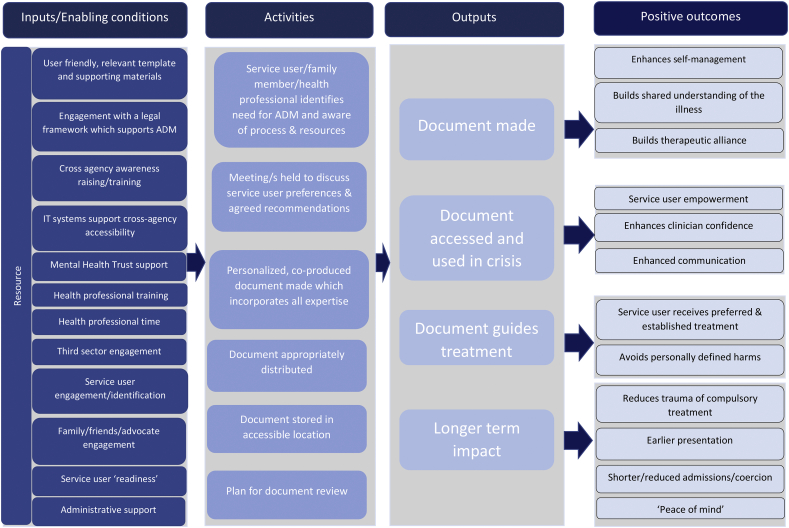
Logic model showing interaction between required inputs/enabling conditions and positive outcomes of Advance Decision Making.

Along with the presentation of the results below, a logic model (Fig. 1) summarises participants' understanding of conditions for enabling successful ADM and desired outcomes. Illustrative quotes are included in the text and detailed for all themes and subthemes in Table 2. Key design dilemmas in creating a single feasible template, guidance and care pathway and how these were resolved during the consultation process are discussed below and summarised in Table 3. The product of this research is the PACT (Preferences and Advance decisions for Crisis and Treatment) - an ADM template with guidance materials (see supplementary materials).

**Table 2:** Quotes from focus group participants relating to themes.

Theme 1: Document form and content	

Subtheme: User-friendly format/language	
Name	*“in terms of the wording ‘self-binding’, it suggests that if something is binding in terms of the contract that some sort of consequence for not following it…. maybe I'm over- thinking it, but I think there are some issues with that wording”* (Consultant Psychiatrist)
Detail vs practicability	*‘my experience with it has made me want to argue for both extremes, for having a very long form, to prompt very fulsome discussion as a therapeutic thing, because I am of the opinion that it can be therapeutic. But on a practical level it has to be short enough for people to actually read in the notes. So maybe that's two different things.’* (Family member)
Structure vs flexibility	*‘I would like lots and lots of different questions to help tease out the….the appropriate and relevant stuff to you.’* (Service User)

Subtheme: Relevant content	
Personalised relapse indicators	*‘So before you take clothes off….a day or two days before….you see that you drink, you smoke, you take drugs, you don't sleep, you eat rubbish food….junk food…. you get upset, you shout at people, you quarrel. All these things are normal …all the normal people do them, but yours will lead to running naked around the streets, singing Marseille’* (Service user).
Facilitating early intervention	*‘there seems to be no intervention until he's at the height of the crisis, whereas there's other symptoms that are lesser….not as detrimental to him… that could be a sign that early intervention is needed, rather than waiting until he's ready to be sectioned.’* (Family member)
Facilitating early compulsory treatment	*“the person that comes to mind that worked very well, he actually wrote on it, ‘I will say and do anything to avoid admission, so just ignore me, and crack on and do your job’….So that works fantastically well”* (AMHP)
Preferences for treatments and care settings	*‘Because I think quite often when people are in crisis, and they're not sort of discharged quite quickly, those are the people that tend to miss out on…accessing psychological groups as an inpatient… I think it's important for that to be kind of mapped out somewhere’* (AMHP)
Crisis contacts	*“many people who when unwell decide that their nearest relative who when well is caring and supportive and understands them, when they're unwell they decide they're poisoning them and they're the cause of all their troubles…So to be able to put in there that ‘Actually this is my nearest relative, and I'm happy for you to contact them, even if I say that they abused me in childhood and poisoned me’. That would be a useful way of us then knowing.”* (AMHP)

Theme 2: Process and context	

Subtheme: Context	
Systemic context	
Legal provision supporting ADM	*‘we need to be just clear with people around the limitations, and obviously where interacts with the Mental Health Act, but unfortunately as it stands the Mental Health Act can trump these directives…. obviously whether that continues in the future, we'll see with the new Mental Health Act.’* (AMHP)
NHS Trust level support	*‘you need to raise it at systemic or strategic level’* (AMHP)
Authentic culture of co-production	*“has to be validity and ownership around it, not just the person writing it taking ownership, but collectively within the environment and the culture that you're working in….that these are actually valid, and people's views are actually heard, rather than it just being an exercise of making you feel better, that ‘When you become unwell we might do this’”* (AMHP)
Limited resource	*‘There's going be things that they will not listen to because …of* (limited) *resources,*’ (Service User)
Fragmented services	*‘if you end up out of borough or you happen to be unlucky in the wrong postcode, your treatment can wildly differ and no one bothers to communicate back to your home postcode.’* (Service User)

Interpersonal context	
Difficulties of discussing coercion	*‘an ideal clinical scenario is that people are actively engaging the patient in all detail, risk and coercion. I think that the sad reality is that those tough conversations don't take place.’* (Consultant Psychiatrist)
Distress	*‘the patient hates recounting their mental history when they think it should be somewhere else. And this is a big problem.’* (Consultant Psychiatrist)
Conflict over treatment recommendations	*‘the reality of the situation that having it written down, that this bit is endorsed by the clinician or not, does set up a lot of conflict in your relationship with the capacitous patient at a time when things are going… potentially going well? … so maybe it just exposes that. That's not to say that you shouldn't….it will bring things up that otherwise people could sort of move on from.’* (Consultant Psychiatrist)
Conflict over defining harm	*‘even when people….have decision-making capacity….that doesn't mean that they're making wise decisions or decisions that the teams are going to be able to adhere to.’* (AMHP)
Undue influence	*‘it later transpired that it (*an ADM document*) had been drawn up between the patient and the mother, with whom there was quite a complicated relationship…. was not clear was to what extent the instructions within it were driven by the mother, or whether they were the genuine preferences of the patient.’* (Care Coordinator)

Personal context	
Acceptance of illness	*‘It can be a difficult diagnosis to accept…So there might be a bit of a lag between someone first having the illness and getting the diagnosis, and actually being able to do this.’* (Family member)
Timing creation of document in illness cycle	*‘doing it when one feels well and better is great, and would be the ideal’* (Service User)
	*‘my best time is always immediately after some sort of big crisis’* (Service User)
Experience of mental health services, including previous compulsory admission	*‘We're all talking about very well-known, well-established people, well-established patterns of illness…. and I think the issue is at what point somebody…. we are clear enough about somebody's patterns of illness and behaviour that this plan would become useful’* (AMHP)
Strongly motivated to avoid harm e.g. in perinatal period	*‘I would say pregnant women, definitely…pregnant women, high functioning women….again it's because they do have a lot to lose.’* (Care Coordinator)

Subtheme: Document review	
*‘the advance directive need not be seen as something which is set in stone, much as it's a very detailed document….there needs to be a review time frame, doesn't there? Because you might find the patient is in a ward setting, having relapsed, thinking to him or herself, “Yes but I did want to change that part of the form but never got round to it.’* (Care Coordinator)	

Theme 3: Accessibility	

Potential to improve access to clinical information	*‘And in theory, could such a document on some computer system that the wards, the A&E, the NHS, the GP, the psychiatrist…everyone can access it?’* (Service user)
Barriers to accessibility	*‘my sort of main concerns are that I'm still convinced that not enough professionals learn from each other or communicate with each other… would it cover enough computer systems?’* (Service User

Theme 4: Harnessing Expertise	

Service User expertise	*‘these people are the expert in their own illness ….you know, and the impact that that has with their life.’* (AHMP)
Family member/friend expertise	*‘We as a family have to try…. and like probe out the psychosis talk, just so they see that he's unwell, otherwise he can easily mask it…otherwise he will go for months where….not quite being sectionable but not being himself, which is very draining and hard.’* (Family member)
Clinician expertise	*‘GPs are very happy with this because it means that they can look and see what the psychiatrists want.’* (Service User)
Combined expertise	*“if there was a crisis coming up and as a team we were aware….the person who'd …co-produced it with them would be able to get that document and…. by sitting down and saying … ‘You've identified these markers, these early warning signs’ ….it was helpful for individuals to realise that something that they'd said earlier and came from them”* (AMHP)

Theme 5: Personalising medico-legal assessment	

On direct engagement with medico-legal framework	*I mean, in general, the actual main thrust of it is more, “I do want it”….it's about sectioning….it's about working with the sort of sectioning process….or working with the….or working with the capacity assessment process, to say, you know “I do accept that actually at times I need this treatment, and I want this document to be part of that….to be part of that assessment process.’ (*AHMP)
Assisting with complex capacity assessments	*‘this form is very useful, and clearly all the case law that's coming along says that we need to take into account people's views when they have capacity, and what is known of them.*’ (AMHP)
Contested capacity	*‘I think by having this document it will help that understanding that ok, he doesn't have capacity and what the family is saying is actually what we've agreed with my dad when he is well.’* (Family member)
Simplifying MHA assessments	*“the person that comes to mind that worked very well, he actually wrote on it, ‘I will say and do anything to avoid admission, so just ignore me, and crack on and do your job’. He actually wrote that….”I'm prepared to say and do anything to avoid admission, so just do your job when I'm that unwell”. So that works fantastically well.”* (AMHP)
De-escalation of crises	*“I know with the self-binding aspect, it would be very helpful for me, because I mentioned sort of having….sort of taking Olanzapine and being sort of ok about that in a crisis. But I know that if I've gone beyond the sort of initial stages, I would refuse Olanzapine because I've….you know, I open up that leaflet and it says one of the side effects, you know, ‘sudden unexplained death’….and it totally freaks me out, and I also think everyone doesn't have my best interests at heart….so it's sort of….it's making it…self-binding for me would be very useful.”* (Service User)
Consequence for stakeholders	*I'm interested in the terminology of self-binding. Because what's the patient binding themselves legally to, rather than just saying this is my wish list? And also, where does it bind the clinicians who sit down and draw this up if it doesn't get enacted?* (Consultant Psychiatrist)

Theme 6: Outcomes of ADM	

Subtheme: Outcomes of making ADM document	
Enhances self-management	*we're all about trying to help patients and their relatives, for patients to self manage. And the more that we can do that we can do that in a focused way, I think, the better* (Consultant Psychiatrist).
Builds shared understanding	*‘the last time I was hospitalised I went in as a voluntary patient, and I was slowly getting to know more about myself…I reckon now is the time that I can be most honest with myself and work out what's going on with myself the most, and be more honest with those nearest to me so that they can pick up the signs’* (Service User)
	*‘it can help families to come together around the illness in a way that we hadn't before we did this kind of process….to realise that my view of what I had seen and understood of her illness was so different from her experience of it. And to come to some kind of shared understanding of it, and understanding the other person's memories of it’ (Family member)*
Builds therapeutic alliance	*I think that the notion about increasing therapeutic relationship is a paramount one. I think it's about not only just the contract the patient is signing, but essentially the Trust is signing with the patient. And the whole endeavour is collaborative from the outset* (Consultant Psychiatrist)
Distress	*‘the patient hates recounting their mental history’* (Consultant Psychiatrist)
Conflict	*‘the reality of the situation that having it written down, that this bit is endorsed by the clinician or not, does set up a lot of conflict in your relationship with the capacitous patient at a time when things are going… potentially going well? … so maybe it just exposes that. That's not to say that you shouldn't….it will bring things up that otherwise people could sort of move on from.’* (Consultant Psychiatrist)
Undue influence	*“There have been huge amounts of pressures from family member sort of saying, you know, ‘Well if you get unwell in the future you're not going to see your kids again’. So, now if that sort of pressure is then on a capacitous person, I can imagine them making a decision that they don't really want to make, because they are, you know, they are being coerced in some shape or form.”* (Consultant Psychiatrist)
	*‘but then people you trust could be manipulating, and use things against you, and relationships break down.’* (Service User)

Subtheme: outcomes of using ADM document during a crisis	
Service user empowerment	*‘We're promoting our patients’ autonomy, they get to make statements about what they want to happen during their next crisis*…’ (Consultant Psychiatrist).
Enhances clinical confidence	*“if you've got that in a document and they did, when they had the capacity, say ‘This is the person I want you to speak to’, then as an AMHP you're going to feel more empowered to speak to that person.”* (AMHP)
Enhances communication	*‘if the advance directive could assist, as a kind of a quicker way to some of the things that actually are helpful and are known to be helpful, instead of each time that somebody gets admitted.’* (Care coordinator)
Concerns that restricts clinical judgement	*‘if it's going to inform a Mental Health Act assessment, in a sense, an early Mental Health Act assessment, then does it undermine the assessor's own thoughts, in a sense, about risk or degree during that assessment’* (Consultant Psychiatrist)
Positives of restricting clinicians	*‘I hate to say they* (Psychiatrists) *were old-fashioned but….I think they were very much of the opinion that….it's for them to decide the treatment. And I think they saw it as them being told by the patient, and people like myself who's not medically trained, the treatment that this patient should be having’* (AMHP)

Subtheme: Outcomes of treatment decisions	
Service user receives preferred & established treatment	*So of course when the person became very ill and they needed to go out….sometimes they were placed in placements very far away…given a number of medications which actually were counterproductive to him becoming well, and actually prolonged and protracted his admission* (Care Coordinator)
Avoid personally defined harms from illness	*‘he has an ingredient as part of the advance directive, that at the point that he's spent this much money within this much amount of time, his bank card gets given to his mum.’* (AMHP)
	*‘she's a working person and she doesn't really want to….in terms of damage limitation to her reputation*’ (Consultant Psychiatrist)
Avoid personally defined harms from treatment	*‘more advance directives was basically…, thinking of ways with him that we could collaborate to reduce the trauma associated with those experiences’* (AMHP talking about a service user experiencing trauma from compulsory detention and treatment)
	*I put on about 11* *kg, which is a lot of weight. Afterwards, you know, trying to get rid of it when you're just not well yourself….so it's hard. Apart from mentally not being well, you stop recognising yourself physically as well* (Service User talking about medication side effects)
Receives sub-optimal treatment	*‘the directive might be completely contrary to NICE guidelines, for example, and I guess….I don't know what the plan would be in terms of…if somebody was putting in an advance directive something that was regarded as potentially quite dangerous for them.’* (Care Coordinator)
Clinician liability	*So the second thing is about how binding it is for the clinician that assesses. And by that I mean, you see a patient, you discharge, commits suicide, goes to coroners, and they say, “Wait a minute. I can see here there was this self-binding directive”… So are they going to be in trouble not following, or are they going to feel, “Oh God, although I do feel that, you know, I could start him on something else, but um….if I do that then I risk my own….”* (Consultant Psychiatrist)

Subtheme: Long term impact	
Reduces trauma of compulsory treatment	*The section on inpatient treatment…. acknowledges that inpatient treatment, particularly if you're sectioned, can be awful, and damaging. And I think that, for a lot of people who've experienced it, they don't feel there is that acknowledgment from staff who've looked after them…And that actually the admission might be something that you have to recover from afterwards*. (Family member)
Earlier presentation	*‘Well I think it empowers people to take a lot of responsibility for keeping themselves well and for…. their seeking out or accepting treatment when they're starting to relapse*.’ (AHMP)
Shorter/reduced admissions/coercion	*‘So it will be useful before they've been sectioned and hopefully instrumental in them not being sectioned, as a way of helping people looking after them to understand the presentation, and to understand how ill they are.’* (Family member)
‘Peace of mind’	*‘it might also provide considerable peace of mind to the client themselves, that this is in black and white, in terms of expression of their own personal agency’* (Care Coordinator)
	*‘I like this concept of legally binding advance decisions, and if I can make it… I would feel safer, and it's wonderful, I think, what you're doing here.’* (Service User)
Disappointment	*‘if you create something that gives people this hope, and then there isn't a provision…. is that even more damaging rather than helpful for them?’* (Consultant Psychiatrist)

**Table 3:** Resolving dilemmas in ADM document, guidance and care pathway design.

Dilemma	Solution	Sources of advice
Misleading name	‘Self binding directive’ changed to ‘PACT’ (Preferences and Advance decisions for Crisis and Treatment)	Problem identified in all focus groups Research team developed name Approved during consultations with service user and professional groups

Detail vs practicability	Adopted a ‘workbook’ style for the body of the main document complimented by final ‘Summary page’ for quick reference in crisis situations. Further explanations about apparent length added to guidance.	Problem identified during Care coordinator and Consultant Psychiatrist focus groups Solution suggested during Consultant Psychiatrist focus group Approved during consultations with service user and professional groups
	Location of mental health history specified on form rather than all information	Problem identified during Care coordinator and Consultant Psychiatrist focus groups Solution suggested in Care coordinator and Consultant Psychiatrist group
	Reduction in legal guidance and content prompts on PACT document whilst retaining full detail in complimentary guidance documents	Problem identified during service user and professional consultation. Balance achieve through iterative process of feedback and refinement during the consultation process

Structure vs flexibility	Structured form with reflective conversation prompts	Need to avoid ‘tick box exercise’ identified by all clinician focus groups Need for structure and prompts identified by Service user focus group Structured preferences and reflective questions developed by research team and approved during all consultations with service user and professional groups

Harnessing the power of clinician endorsement vs authentically representing service user wishes	Advance preferences and requests structured according to the following categories: • Service user preferences with prompts of explanation • Agreed recommendations • Comment boxes for health professional available to endorse service user preferences or raise concerns	Concern about power dynamics raised in Service User and AMHP focus group Extensive discussion about managing power imbalances within the research team Solution developed by research team Approved during consultations with service user and professional groups
	Guidance on legal implications and non-necessity of clinician endorsement of ADRT clarified	

Potential for discrepancy between legally (MHA) defined ‘nearest relative’ and preferred crisis contact	Section to document ‘nearest relative’ plus section for service user to specify preferred crisis contacts plus those they would prefer were not contacted	Identified during legal consultation and AMHP focus group Solution discussed amongst research team Approved during consultations with service user and professional groups

Potential to bring ‘peace of mind vs potential to cause distress and disappointment	Explicit discussion of potential for process to cause distress in guidance document Guidance included on the clinical and legal limitations of the document Guidance given around creating supportive meeting environment and process for creating document	Advice in guidance document based on input from Mental Health and Justice Service User Advisory Group

Potential to build alliance during process of making document vs potential for conflict and undue influence	Questions on template designed to prompt whole group reflection Preferences section structured to allow for difference of opinion without losing jointly agreed treatment recommendations Guidance documents emphasise the importance of all voices being heard during discussions and documenting perspectives raised	Problem identified in all focus groups Idea for reflective questions developed within research team Phrasing reflective questions discussed with Consultant Family Therapist with expertise in Open Dialogue approaches to mental health crises Guidance document content informed by consultation with service user led organisations

Potential to enhance quality of clinical decision making vs concerns about clinician liability	Explicit guidance on legal status of document on template and in guidance, including documentation of rationale for deviating from contents of document	Problem identified in Consultant Psychiatrist focus groups Potential for clinician liability discussed during legal consultation and resultant advice used to inform guidance

Ensuring accessibility vs protecting confidentiality	Section on the form to prompt discussions around storage plan including preference around who has a copy Suggestions for ensuring accessibility in crisis included in guidance documents Confidential nature of document explicit on template	Problem identified in all focus groups Ideas around ensuring accessibility offered in all focus groups Potential solutions collated and included in guidance documents by research team Approved during consultations with service user and professional groups

Respecting advance personalised medico-legal assessments and contemporaneous clinical judgement	Structured prompts on template to ensure information relevant to MCA/MHA assessment is clear for future assessors Guidance on legal implications in template and guidance documents	Issue raised during legal consultation and Consultant Psychiatrist focus group Guidance around legal components written based on results of legal consultation and discussion within research team Approved during consultations with service user and professional groups

Standardised care pathway for document creation vs allowing for individual needs	Suggested care pathway included in guidance documents Explicit that the length of time/number of meetings each service user requires to complete document may vary	Ideas for a care pathway discussed in focus groups Research team developed draft care pathway Input for MHJ SUAG refined care pathway Approved during consultations with service user and professional groups

### 3.1 Theme 1: template form and content

All participants identified format and language of materials as fundamental to the success or failure of ADM documents (subtheme). Key dilemmas involved naming the document, balancing the need for detail with manageable length and providing structured response prompts while avoiding becoming a ‘tick box exercise’.

Participants were keen to include elements of informal crisis planning, the usual form of mental health ADM, while ensuring that information identifying relapse indicators and the recommended response is highly personalised.

Use of ADM documents to express treatment requests relevant to *all* stages of an episode, from early crisis through to discharge from hospital was less familiar but universally supported. While the most common category of preferences concerned particular medications, the form also included space for preferences around medical and non-medical treatments and relevant care settings: community/home treatment teams, inpatient admission and discharge.

There was enthusiasm about personalising crisis communication preferences by changing the legally defined ‘nearest relative’ to the service user's ‘nominated person’, in line with expected MHA reform.

### 3.2 Theme 2: process and context

All participants saw making and using ADM documents as a reflective process rather than single event, with a built in review period (subtheme). Creating co-produced ADM documents was thought to require a series of thoughtful conversations with authentic engagement from all parties.

*“has to be validity and ownership around it, not just the person writing it taking ownership, but collectively within the environment and the culture that you're working in….that these are actually valid, and people's views are actually heard, rather than it just being an exercise of making you feel better, that ‘When you become unwell we might do this’”* (AMHP).

Context was seen as critical to enabling quality of this process at three levels. First, the wider systemic context (subtheme), including legal frameworks, resources available (both to create documents and meet treatment preferences), NHS Trust level support and authentic clinical openness to co-production. Second, the nature of the interpersonal relationships (subtheme) between those making the document, given potential difficulties of discussing coercion, concern about causing distress, conflict over treatment recommendations or harm definitions and preventing undue influence from e.g. family members. Third, the service user's personal context (subtheme): their past treatment experiences, acceptance of illness, motivation to avoid particular harms, and ensuring documents are created when sufficiently recovered from recent crisis.

Addressing concerns around interpersonal power differentials within the drafting process proved a significant challenge, especially the question of how to allow for the potential added ‘clout’ that including clinician endorsement of service user treatment preferences might bring without devaluing non-endorsed preferences. A further complication was finding language which made clinicians comfortable with the responsibility involved in endorsing advance requests and commenting on refusals. The solution was to include distinct sections for service user requests, agreed requests and for each party to explain rationales. Signatures from all parties to affirm accuracy of discussion summaries was also requested.

### 3.3 Theme 3: accessibility

A major universal concern was ensuring clinician awareness of existing documents and accessibility, particularly for someone presenting out of area or lacking social support. More positively, many saw ADM documents as having potential to improve cross-agency communication and hoped that checking records for ADM documents could become standard procedure, particularly considering expected MHA reforms.

*‘I'm still convinced that not enough professionals learn from each other or communicate with each other and that this, depending on if you can get it onto some computer systems… would it cover enough computer systems?’* (Service User).

A ‘PACT access plan’ section was introduced to help devise appropriate individualised storage plans, enabling accessibility. Confidentiality concerns were resolved by highlighting the confidential nature of the document on each page and including a declaration of permission for the document to be seen and used by professionals.

### 3.4 Theme 4: harnessing expertise

An idea highlighted repeatedly was the importance of ‘harnessing expertise’ from ‘lived experience’ to produce the document. Cyclical and often highly repetitive illness patterns create knowledge which can help inform future treatment decisions, a phenomenon unique to clinical contexts with marked and predictable episodes of illness and fluctuations in DMC. The reflective questions in the PACT aim to encourage service users to adopt a position of hindsight and draw out their expertise.

### 3.5 Theme 5: personalising medico-legal assessment

The idea of direct service user engagement with the medico-legal framework was received positively and seen as a valuable distinguishing factor of the prototype PACT document.

*‘this form is very useful, and clearly all the case law that's coming along says that we need to take into account people's views when they have capacity, and what is known of them……it gives you valid reasons for setting aside some of the things they may be saying which appears to give capacity…certainly the judges are telling us through their case law that we should be taking these sorts of forms into account.*’ (AMHP).

Given that DMC-T assessment, especially in early crisis, was widely viewed as potentially complex and contested, a personalised capacity assessment component was introduced to help manage such difficulties.

*‘I think by having this document it will help that understanding…he doesn't have capacity and what the family is saying is actually what we've agreed with my dad when he is well. And that would help a lot.’* (Family member).

Clinicians recognised the document's potential to increase confidence in detecting subtle signs of loss of DMC-T or managing discrepancies between expressed and enacted DMC-T.

Participants welcomed using the form to specify preferred conditions for MHA assessments. Determining thresholds for coercive treatment to manage risk was an emotive issue. Nevertheless, all participants saw a role for ADM in risk avoidance, despite likely disagreement about what constituted the kind of risk demanding intervention. While some participants assumed avoidance of admission as the main aim, there was also significant interest in using ADM to prevent harm through requesting treatment under the MHA, this type of arrangement is sometimes known as a ‘self-binding directive’. In response the template was worded to encourage discussions around personalised definition of harms across multiple domains.

Some clinicians recounted positive experiences of how such ‘self-binding directives’ make MHA assessments less traumatic for service users and more straightforward.

*“‘I will say and do anything to avoid admission, so just ignore me, and crack on and do your job’. He actually wrote that….So that works fantastically well.”* (AMHP).

In response to some concerns about uncertainty over the legal status of the ADM document guidance was included, drafted in collaboration with legal experts.

### 3.6 Theme 6: outcomes

A diverse range of potential outcomes, both positive and negative, were envisaged by all parties and understood to occur at multiple points during the process of making and using ADM documents. Participants' experience led them to believe the process of creating these documents could offer a space for service users to reflect on their experience of living with their illness leading to enhanced self-management. In addition, it was felt that a collaborative process of making ADM documents could increase understanding of the service user's experience and foster a stronger therapeutic alliance with family members and health professionals.

*‘the last time I was hospitalised I went in as a voluntary patient, and I was slowly getting to know more about myself…I reckon now is the time that I can be most honest with myself and work out what's going on with myself the most, and be more honest with those nearest to me so that they can pick up the signs’* (Service User).

*‘it can help families to come together around the illness in a way that we hadn't before we did this kind of process….to realise that my view of what I had seen and understood of her illness was so different from her experience of it. And to come to some kind of shared understanding of it, and understanding the other person's memories of it’* (Family member).

Conversely, (as discussed in section 3.2) there was a concern that reflecting on past traumatic experiences may be distressing and that a collaborative approach may potentiate conflict where there is disagreement about treatment options and undue influence.

If ADM documents are accessed in a crisis, participants believed this would be empowering for service users and clinicians in that they would help communicate high quality information and guide confident clinical decision making.

*‘if the advance directive could assist, as a kind of a quicker way to some of the things that actually are helpful and are known to be helpful, instead of each time that somebody gets admitted.’* (Care coordinator).

If the contents of the document were applied to inform treatment choices participants saw the potential for service users to receive preferred and established treatment and avoid personally defined harms from the illness and unsuitable care and treatment.

Although the advantages of informing clinical decision making were well understood there was some concern, particularly amongst psychiatrists, that their decision making may be undermined, service users would receive sub optimal treatment and that as psychiatrists they could be liable. In response, clarity on this issue was sought during legal consultation and included in the guidance documents.

Participants did reflect on the impact ADM documents might have on compulsory admission to hospital. Some hoped they may encourage early help seeking and thus de-escalation of crises removing the need for admission. Others hoped they could trigger early intervention and facilitate a shorter, less traumatic compulsory admission. In turn, this could improve the service user's overall mental health.

*‘The section on inpatient treatment…. acknowledges that inpatient treatment, particularly if you're sectioned, can be awful, and damaging. And I think that, for a lot of people who've experienced it, they don't feel there is that acknowledgment from staff who've looked after them…And that actually the admission might be something that you have to recover from afterwards*.’ (Family member).

In the longer term service users hoped using ADM documents could offer ‘peace of mind’ that they could be meaningfully involved in shaping a reliable and helpful response to a crisis.

*‘I like this concept of legally binding advance decisions, and if I can make it… I would feel safer, and it's wonderful’* (Service User).

Health professionals also hoped for this outcome but expressed concern that if service users were not able to make and use these documents within a trustworthy system the disappointment they may experience if their documents were not accessed or dismissed could be damaging.

*‘if you create something that gives people this hope, and then there isn't a provision…. is that even more damaging rather than helpful for them?’* (Consultant Psychiatrist).

Service users and service user organisations were consulted about this point and agreed there was a potential for individuals to experience painful disappointment. However, they advised this possibility should not be a barrier to encouraging engagement in ADM but highlighted the importance of statutory reforms in promoting the status of ADM documents and the necessity of providing support and high quality information about their clinical and legal limits.

## 4 Discussion

### 4.1 Summary of main findings

We identified a diverse range of outcomes and clarified understanding of enabling conditions and barriers for mental health ADM. Taking account of such factors, we have co-produced an ADM document, the PACT (Preferences and Advance decisions for Crisis and Treatment), a care pathway and guidance documents (see supplementary materials).

### 4.2 ‘PACTs’ compared to other models of advance decision making

Mental health ADM has shifted greatly from early associations with the ‘anti-psychiatry’ movement and complete refusals of treatment (Szasz, 1982). Internationally, a large variety of tools and processes (Henderson, Swanson, Szmukler, Thornicroft, & Zinkler, 2008; Owen et al., 2019) now allow service users to make treatment *requests* and recommendations, alongside more specific *refusals*. The UK's most extensively researched model, the Joint Crisis Plan (JCP) supports facilitated, collaborative, informal ADM with a primary aim of reducing compulsory admission. Success in a small early trial of JCPs (Henderson et al., 2004) was not replicated in a larger trial, possibly due to reduced clinical engagement (Farrelly et al., 2016, Thornicroft et al., 2013). Similar studies on JCPs in the Netherlands (Ruchlewska et al., 2014) and Switzerland have also had some success (Lay et al., 2015)(). PACTs may build on the success of JCPs by offering supported formal ADM, within an environment where statutory reform may make clinician engagement more likely.

PACTs contain service user requests and refusals (rather than relying on proxy decision makers) and support collaborative decision making. They have some distinctive characteristics, especially their explicit and guided engagement with medico-legal frameworks, designed to cater for fluctuating capacity within SMI. The PACT is a model with explicit space and guidance for personalised input into medico-legal assessment, while also allowing ‘authentication’ of the document itself, through elements such as documented assessment of capacity when drafting and specification of review period. They are also designed to challenge the model of ADM as primarily a tool to avoid compulsory treatment (de Jong et al., 2016; Molyneaux et al., 2019), by identifying and faciliating diverse outcomes; building, for example, on law reform supporting ‘self-binding’ in jurisdictions such as the Netherlands and Washington State (Berghmans & van der Zanden, 2012; Varekamp, 2004).

PACTs also take lessons from the US, where legal provision is more widely established (Van Dorn, Swanson, Swartz, Elbogen, & Ferron, 2008) yet studies consistently demonstrate disappointingly low levels of document completion, despite high levels of service user demand (Srebnik, Russo, Sage, Peto, & Zick, 2003; Swanson et al., 2006b; Swanson et al., 2003). Possible explanations from research in the US include difficulties within the documents and lack of adequate support (Srebnik & Brodoff, 2003; Van Dorn, Swanson, Swartz, Elbogen, & Ferron, 2008), with one RCT showing facilitation to be effective in increasing uptake (Swanson et al., 2006a). Therefore, while PACTs are clearly structured around legal provision, they are also designed as flexible documents, to be made collaboratively, with clinical and family support and with clear guidance available for all parties. By treading a flexible middle ground between minimal and highly intensive clinical support, PACTs may help to achieve the benefits of ADM whilst remaining feasible within resource constrained clinical services.

### 4.3 Strengths and limitations

A major strength of this study was consultation with a wide range of stakeholders who would be directly involved in making and using ADM documents. In addition, data was collected and analysed by researchers with diverse academic, professional and lived experience. Limitations include that, despite our efforts, not all service users recruited for focus groups had lived experience of MHA assessment and detention. Nevertheless, even these service users saw an ADM document as personally relevant. Our sampling with regard to fluctuating capacity was restricted to bipolar though this gave us more confidence that our user focus groups were experts by experience in fluctuating capacity. Given that most (but not all) participants came from London, there may also be uncertainties regarding wider generalisability.

### 4.4 Future directions

In terms of future directions, the PACT materials, underpinned by increased understanding of how to enable ADM with diverse outcomes, pave the way for further evaluation of process and outcomes of implementation within mental health settings. This is crucial, given the likely MHA reforms will apply across all clinical settings. A regional project - CPMS 43078 - PACTs for Bipolar - is exploring the experience of service users, their family members and treating clinicians in South London and Maudsley NHS Foundation Trust. Projects using the PACT in other regions of England or other jurisdictions (e.g. USA or Europe) may also be possible with suitable adjustments. Early results of this study suggests that the activity of making high quality, collaborative ADM documents could be difficult to absorb within the typical structure and resources of current mental health settings e.g. community mental health teams. One way to anticipate and make provision for ADM could be dedicated ‘clinics’ or ‘workshops’, which interface with local service user organisations and clinical teams. Such a clinic could also provide a hub within regional mental health, able to lead on education, training and increasing access. This may include using technology to develop online resources and remote consultation. Research linked to such a clinic could clarify the ways in which PACTs need to be adapted for those who have different experiences of mental illness, of losing DMC-T and of mental health services. For example, those who have a high level of trust in mental health services vs those who have had traumatic experiences. While PACTs are designed for those who have a fluctuating pattern of DMC-T, there may be service users who do not regain DMC-T between episodes of illness and still wish to express their wishes about treatment. Further work could involve co-producing resources to support collaborative ADM in this group to ensure that all who wish to engage in mental health ADM have options to participate.

## 5 Conclusions

Desired aims for ADM catering for fluctuating capacity within a mental health context may range from avoidance to facilitation of admission and include both requests and refusals. This needs clear and guided personalised engagement with medico-legal processes. To achieve this, The PACT ADM template and guidance materials have been co-produced and assessed for feasibility through extensive and innovative consultation and review with key stakeholders. We are making them available, in response to increasing demand from individuals and organisations, and also hope to stimulate further research, development and availability of mental health ADM resources and optimise future implementation.

## Funding

This work was funded by the 10.13039/100010269Wellcome Trust, United Kingdom– grant number 203376/Z/16/Z.

## Declaration of Competing Interest

None.

Alex Ruck Keene was the legal adviser to the Independent Review of the Mental Health Act.

## Appendix A Supplementary data

PACT Template Image 1

Service User PACT Guidance Notes Image 2

Health Professional PACT Guidance Notes Supplementary material 3 Image 3

## Footnotes

2 A full discussion of this issue can be found in this report: (Martin et al., 2016)
