# Supplementary material for: The PACT advance decision-making template: preparing for Mental Health Act reforms with co-production, focus groups and consultation
Source: Int J Law Psychiatry. 2020 Jul-Aug;71:101563. doi: 10.1016/j.ijlp.2020.101563 (PMC7435693; doi:10.1016/j.ijlp.2020.101563)
Supplement: Supplementary file 1 — PACT Template [file mmc1.pdf]

PACT

Preferences and Advance decisions for Crisis and Treatment

Name:

Mental Health and Justice

King's College London

Contents

Contents	Section
Information for health professionals providing me with care	
**Information about me**	
Personal Details	1
Signs that I am becoming unwell	2
Key risks	3
Information for health professionals completing a mental capacity assessment	4
Information for health professionals completing a Mental Health Act assessment	5
Key crisis contacts	6
**Legal documents:** **Advance statements of wishes and feelings and Advance Decision to Refuse Treatment**	
Confirmation of mental capacity to complete PACT	7
Advance statement of wishes and feelings about community/ Home Treatment Team care	8
Advance statement of wishes and feelings about the care I receive if I am admitted to hospital	9
Advance statement of wishes and feelings about non-medical treatments	10
Advance statement of wishes and feelings about medical treatments	11
Advance Decision to Refuse Treatments	12
**Administrative information**	
PACT review plan	13
PACT access plan	14
Others involved in making this PACT	15
**Summary**	
PACT summary page *For use when quick reference is required in a crisis*	Final page

Information for health professionals providing me with care

Health professionals who are providing me with care should read this document.

This document is my 'PACT'. PACT stands for Preferences and Advance decisions for Crisis and Treatment. It is combines information that you might find in a mental health crisis plan with two legal documents that are described in the Mental Capacity Act 2005 (MCA): an Advance Statement of wishes and feelings and an Advance Decision to Refuse Treatment.

My PACT has been designed to help health professionals provide me with care if I lose the mental capacity to make decisions about my treatment. It has been made with health professionals and other people I trust at a time when I was assessed as having the mental capacity to make my own decisions about treatment for mental health problems.

The legal documents contained in the PACT are summarised in the table below:

Decision	Legal name for this type of decision (according to the Mental Capacity Act 2005)
Treatments I want/would prefer	Advance Statements of Wishes and Feelings
	Advance Statements of Wishes and Feelings containing decisions which have been shared and agreed with a health professional
Treatments I do not want	Advance Decisions to Refuse Treatments

I know that not all my decisions and preferences may be followed in a crisis, particularly if I am admitted to hospital under the Mental Health Act 1983. However, if my decisions and preferences are not followed, and particularly those that have been shared and agreed in advance with health professionals, the reasons should be written down in my medical notes.

I am willing for my PACT to be available on my confidential clinical care records and for it to be accessible to all professionals involved in my care.

Information about me

Section 1: Personal Details

Name
(Include full name, preferred name, previous names and aliases)

Date of birth

NHS number

Main mental health problems
(Could include main current diagnoses on health records, service user and/or health professional description of mental health problems)

Details of my mental health history can be found at the following location:
(e.g. Mental Health Trust records, GP records, other location)

Section 2: Signs that I am becoming unwell

Discussion ideas
If someone I trust were to try and describe me when I was becoming unwell, they would say...

Answer ideas

Type of sign	Example of sign
Change in beliefs	Unusual beliefs about special powers/abilities, feeling paranoid, different attitudes to loved ones
Change in behaviours	Spending money unusually, change in activity levels, using alcohol or other substances differently
Change in speech	Speaking faster/slower than usual, difficult to understand, unusual words

Section 3: Key risks

Discussion ideas
When I think I am going to become unwell again the things I worry about the most are...

Answer ideas

Type of sign	Example of sign
To myself	Self-harm, over-spending, damaging my property, impact on my job, social media use
To others	Being less able to care for the people I usually look after, upsetting or hurting others
From others	Getting into embarrassing or dangerous situations

Section 4: Information for professionals completing a mental capacity assessment

(Note to service users completing this form: for more information on what 'mental capacity' means please see the PACT Service Users' guidance leaflet)

(Note to health professionals using this form in crisis: this information should be used to inform assessment of whether the service user's mental health problem is impacting their mental capacity to make decisions about treatment for their mental health problem. For further guidance see sections 1-3 of Mental Capacity Act 2005 and Mental Capacity Act Code of Practice)

Discussion ideas	
The advice I would like to give to the person who will be assessing my capacity to make decisions about my treatment is...<br>If I were to ask someone I trust how they know when I have lost the capacity to make decisions about my treatment, they would say...	
**Answer ideas**	
**Type of sign**	**Example of sign**
Change in beliefs	Change in beliefs about medication, treatment, the need to go to hospital
Change in behaviour	Change in willingness to work with professionals
Change in speech	Typical things I might say

Signs that I may have lost my capacity to make decisions about mental health treatment (and that I need others involved)

Signs that I may have regained my capacity to make decisions about mental health treatment

Section 5: Information for professionals completing a Mental Health Act assessment

(Note to health professionals using this form in crisis: this information along with the information on 'Key risks' should be used to inform assessments of the 'nature' and 'degree' of a service user's mental disorder and whether compulsory inpatient admission and treatment is 'warranted'.)

Discussion ideas	Answer ideas
If I were to ask someone I trust how they would tell if I needed a Mental Health Act assessment, they would say…	I would prefer to be assessed in a clinic/in my home
If it were possible for me to have a Mental Health Act assessment which went well this is what would happen…	If you believe forced entry to my home is required, please consider these options…
If I could give advice to people involved in any future Mental Health Act assessments, I would say…	Hospital admission can help me avoid risks like…

Signs that I may need a Mental Health Act assessment and inpatient treatment

Nearest relative in the event of a Mental Health Act Assessment

Preferred contact in the case of a Mental Health Act Assessment
(If you are not happy for your nearest relative to be contacted if you are going to be assessed, who would you prefer to be contacted and why?)

Section 6: Key crisis contacts

Answer ideas				
Family members	Friends	GP	Psychiatrist	Care coordinator
Social worker	Other medical teams	Tutor	Employer	Bank

In a crisis, the following people should be informed

Name	Role	Information that should be given	Contact details

In a crisis, I would prefer that the following people were not informed/the following information is not shared

Legal documents: Advance statements of wishes and feelings and Advance Decision to Refuse Treatment

Section 7: Confirmation of mental capacity to complete PACT

I confirm that ………………………………… has the mental capacity to make the following Advance Statements of wishes and feelings and Advance Decisions to Refuse Treatments.

My views on the content of these Advance Statements and Advance Decisions to Refuse Treatments are set out separately in each section.

Name of clinician	Role	Signature	Date

Signature required

Section 8: Advance statement of wishes and feelings about community/ Home Treatment Team care

Discussion ideas:	Answer ideas:
I have felt helped through a crisis by a community team/Home Treatment Team when they did...<br><br>If I were to ask someone I trust when they thought I needed a Home Treatment Team, they would say...	Helpful questions people asked me are...<br><br>Helpful ways people found to offer me medication...<br><br>Other services I have found helpful in a crisis are...

a) Community/Home Treatment Team care I want/would prefer and why

(Note to service users completing this form: fill in this box before meeting a health professional to discuss your preferences)

b) Community/Home Treatment Team care we agree should be given in a crisis

(Note to service users and health professionals completing this form: fill in this box together during the meeting to discuss the service users' preferences)

c) Health professional comments

Why I agree/have concerns

(This box should be completed by the health professional)

Section 9: Advance statement of wishes and feelings about the care I receive if I am admitted to hospital

Discussion ideas:

If I were to have an admission which I could look back on as being overall helpful for me this would have happened...

You will know my mental health is improving when I...

Answer ideas:

Personal arrangements if I am admitted to hospital	Support from ward staff	Managing compulsory treatment	Discharge Planning
• Caring responsibilities e.g. children/vulnerable adults/pets<br>• Financial arrangements e.g. bill payments collection of benefits/pension<br>• Property security	• Ward staff could support me by...<br>• Staff could help me if I am anxious/angry/frustrated/upset by...<br>• If a ward round were to be helpful for me those involved would...	• If I need medication but am refusing the best way to talk to me about it would be...<br>• If I have to be restrained, I would like staff to be aware of...	• If my discharge plan was successful and I carried on getting well after I left hospital it would include... (e.g. loved ones/services)

a) Care I want/would prefer to receive if I amadmitted to hospital and why

(Note to service users completing this form: fill in this box before meeting a health professional to discuss your preferences)

b) Care we agree should be given during a hospital admission

(Note to service users and health professionals completing this form: fill in this box together during the meeting to discuss the service users' preferences)

c) Health professional comments

Why I agree/have concerns

(This box should be completed by the health professional)

Section 10: Advance statements of wishes and feelings about non-medical treatments

Examples of non-medical treatments: *Talking therapies/exercise/yoga/meditation/creative activities/diet*

Discussion ideas:	Answer ideas:
I have felt at my healthiest when I...	Looking back, the following non-medical treatments have been helpful to me in a crisis...<br><br>When I was last admitted this non-medical treatment made a difference to me...<br><br>During my last admission I liked to use my time away from the ward in this way...

a) Non-medical treatments I want/would prefer and why

(Note to service users completing this form: fill in this box before meeting a health professional to discuss your preferences)

b) Non-medical treatments we agree should be given in a crisis

(Note to service users and health professionals completing this form: fill in this box together during the meeting to discuss the service users' preferences)

c) Health professional comments

Why I agree/have concerns

(This box should be completed by the health professional)

Section 11: Advance statements of wishes and feelings about medical treatments

Examples of medical treatments: *Medications/ECT*

Discussion ideas:	Answer ideas:
I know a medicine is, overall, helpful/ unhelpful for me when I feel...	I want this treatment in this way (e.g. table, liquid, injection)
For me, the most important side effects to avoid are...	I am willing to take this medication if necessary
My opinion about ECT is...	I would prefer to avoid this medication but will take it if there is no other option

a) Medical treatments I want/would prefer and why

(Note to service users completing this form: fill in this box before meeting a health professional to discuss your preferences)

b) Medical treatments we agree should be given in a crisis

(Note to service users and health professionals completing this form: fill in this box together during the meeting to discuss the service users' preferences)

c) Health professional comments

Why I agree/have concerns

(This box should be completed by the health professional)

I confirm I took part in the discussions about the advance statements of wishes and feelings set out above and what is written is a fair summary of these discussions

Service user name and signature	Date	Health professional name, role and signature
Service user name and role		Health professional name and role

Signature required

Signature required

Section 12: Advance Decision to Refuse Treatment

(Note to service users and health professionals completing this form: this form should not be used to document refusals of life-sustaining treatment)

Medical Treatment that I do not want

(Note to service user completing this document: Include reasons why you want to refuse some medical treatments, for example some medications or ECT)

Signature required

Service user signature	Date

c) Health professional comments

Why I agree/have concerns

Health professional name, role and signature	Date
Health professional name and role	

Signature required

Administrative information

Section 13: PACT review plan

(Note to health professionals using this form in crisis: if the review period has expired this document is still valid but care should be taken to ensure no more recent version is available and that it is still applicable to the current situation)

Answer ideas:
After a crisis/after 1 year/after 3 years/after any significant life event

I will review my PACT…

Section 14: PACT access plan

Copies of this document are accessible in the following locations or with the following individuals

Section 15: Others involved in making this PACT

Name	Role	Signature	Date	Contact details

Spare page

Note to service users and health professionals completing this form: this page can be used for any extra information you wish to include

PACT summary page

(Note to service users and health professionals completing this form: fill out the PACT summary last, using the information from the rest of the form)

Key risks I want to avoid *(for details see section 3)*

The most important information for people assessing me in a crisis to be aware of is...
(for details on mental capacity assessment see section 4, for details on Mental Health Act assessment see section 5)

Treatment I want that I have made a decision about with a health professional *(for details see sections 8/9/10/11)*

Treatment I do not want *(Advance Decision to Refuse Treatment, for details see section 11)*
