# Supplementary material for: The PACT advance decision-making template: preparing for Mental Health Act reforms with co-production, focus groups and consultation
Source: Int J Law Psychiatry. 2020 Jul-Aug;71:101563. doi: 10.1016/j.ijlp.2020.101563 (PMC7435693; doi:10.1016/j.ijlp.2020.101563)
Supplement: Supplementary file 2 — Service User PACT Guidance Notes [file mmc2.pdf]

How to make and use a PACT
(Preferences and Advance decisions for Crisis and Treatment)

What is a PACT?

PACT stands for Preferences and Advance decisions for Crisis and Treatment. It combines the kind of information you often find in a mental health **crisis plans** with two documents that are described in a law called the Mental Capacity Act 2005: an **advance statement** and an **advance decision to refuse treatment**.

PACT forms and this guidance were developed following research with people who are living with mental illness, family members of people with severe mental illness, psychiatrists, specialist social workers, lawyers and care coordinators.

Why should I make a PACT?

Many people with mental health problems who have experienced several mental health crises find they become experts in knowing what are helpful and unhelpful things to happen to them when they become unwell. PACTs are designed to use your expert knowledge of managing your mental health in combination with ideas from family/friends and trusted health professionals (doctors, nurses, psychiatrists, social workers, care coordinators) to make a clear guidance for what should happen if you become unwell in the future.

Other people who have been involved in making documents like a PACT have said:

'it can help families to come together around the illness'
(Family member of someone who has bipolar)

'it empowers people to take a lot of responsibility for keeping themselves well and ….. their seeking out or accepting treatment when they're starting to relapse'
(Social worker who specialises in mental health)

When we asked people who have a mental illness about some of the problems they have experienced trying to get the right sort of help during crises they talked about:

- Recognising your own signs of relapse
- Getting medication that is helpful in a crisis and having support taking that medication
- Making sure the right people get told about the crisis, especially trusted family members/friends
- Communication between health teams e.g. GP, social services, mental health teams
- Getting support from a Home Treatment Team (crisis team) at the right time
- Having more say in how legal assessments are made, for example Mental Capacity Act assessments or Mental Health Act assessments (getting 'sectioned')

- Getting admitted to hospital at the right time
- Having more say in how compulsory treatment (e.g. injections of medicine or seclusion) might be avoided or if it cannot be avoided how it can be best managed
- How health teams might make sure the right sort of support is available on discharge from hospital
- Dealing with health professionals you don't know when you are unwell

PACTs are designed to work with the current laws that apply to how people experiencing mental health crises are treated (the Mental Capacity Act 2005 and the Mental Health Act 1983) to help make sure the person experiencing the crisis keeps as much control as possible over what happens to them. The idea is that, even if the health professionals who are treating you in a crisis haven't met you or the team/health professional who knows you best, they can use the PACT to better understand what works for you when you are unwell.

'*normally it's my word against a clinician, you know, and that's more problematic, particularly if you don't have a relationship with them, and often one doesn't'*
(Service User)

What is in a PACT?

A PACT combines 3 types of document:

1. A crisis plan
   This has information about your mental health problems and how you and other people you trust might know you are starting to become unwell (relapse indicators). We have included a section for you to give information to professionals that will mean they could carry out a ***personalised* mental capacity assessment** and a ***personalised* mental health act assessment**.

   What is a personalised mental capacity assessment?
   When you make your PACT there is an opportunity to discuss with your clinician and friend/family member the kinds of things you say and do when you are losing the mental capacity to make decisions about your care and treatment. If you have a crisis this means professionals can look at your PACT and have more of an idea of whether your decision-making is being impacted by your mental illness or not.

   What is a personalised mental health act assessment?
   When you make your PACT there is an opportunity to discuss with your clinician and friend/family member the kinds of situations when a Mental Health Act assessment (sectioning) might be needed. This may be when you become so unwell that there might be a risk that you say or do things that could cause harm to yourself or other people. Your PACT would contain information on what *you* and your family/friends consider to be harmful behaviour and what can be done to reduce this harm.

2. A written advance statement of wishes and feelings
   Your PACT has sections where you can write your preferences and requests for where and how you are treated and what kinds of treatments you have. In law, under the Mental Capacity Act, this is known as an **advance statement.**

3. An Advance Decision to Refuse Treatment
   This section of the PACT allows you to name medical treatments for mental health problems (e.g. medications or ECT) that you would not like to be given in a crisis.

There is more information on how to fill in these sections of your PACT below.

Is my PACT legally binding?

This table shows which parts of your PACT are legally binding i.e. which parts the law says mental health services have to follow:

What type of document?	Which law?	Is it legally binding?
Advance statement of wishes and feelings	Mental Capacity Act 2005	No
Advance decision to refuse treatment	Mental Capacity Act 2005	Yes<br>Unless you are admitted to hospital under the Mental Health Act. Doctors and nurses do not legally have to follow your advance decision to refuse treatment. But there is a code of practice which advises them they should whenever possible.

The Mental Capacity Act says that you have the right in advance of a time when you might lose the ability to make that decision for yourself (e.g. during a mental health crisis) to state which specific medical treatments (e.g. medication) you do not want to be given. Health teams would then not be legally allowed to give you that specific treatment. However, if somebody is admitted to hospital under the Mental Health Act (sectioned) this no longer applies and they can be given medical treatments for mental disorder, even if they have said in advance they do not want to have them. This is a bit different for ECT. If somebody has made an advance decision to refuse ECT and then is admitted to hospital under the Mental Health Act they can only be given this treatment if their life would be in danger if they did not have it.

However, the official guidance for health professionals using the Mental Health Act says that patient’s wishes, advance statements and advance decisions to refuse treatments should be taken into consideration and followed whenever possible. So, it is still important to document your wishes and preferences and clinicians may be very appreciative that you have done so to help them make the best decisions.

How should I make my PACT?

Making a PACT might take quite a long time. You and your family/friends and health team might have to think about it carefully and meet to discuss what goes into the PACT. Below is a diagram which shows one way you could go about making your PACT.

Interested in making a PACT?

You ask a mental health professional (psychiatrist, care coordinator, psychologist, OT, nurse) about making a PACT or they suggest you might be interested

- You are given the PACT pack (template and guidance)
- You arrange your first meeting to discuss your thoughts on what you might write in your PACT
- You show the PACT to trusted family members/friends/supporters and discuss your ideas

↓

Getting ideas for your PACT

Meeting with one health professional (e.g. your care coordinator):

- Look through PACT template
- Discuss any unclear sections
- Start writing your PACT
- Date of second meeting arranged
- Encouraged to discuss further with family and friends

↓

Once you have written down as many early thoughts as you can, send a copy to your clinical team/the people you are making your PACT with

↓

Making your PACT

Meeting with those involved e.g.: psychiatrist/responsible clinician, care coordinator, family/friends/advocate

- Everyone discusses what is written in your PACT and adds their ideas
- Talk about any disagreements
- Write down how you will store your PACT and who will have access to it
- Write down when your PACT should be reviewed

↓

Cooling off period

- Look through your PACT after the meeting and think through whether there is anything you want to change
- If you have any concerns another meeting can be arranged

Taking part in a PACT meeting

PACTs are designed to be made between you and a trusted health professional it can include any other trusted person such as a family member/friend/peer support/advocate. One of the purposes of the PACT form is to provide ‘conversation starters’ for people taking part in the PACT meeting to discuss. We think the best PACT meetings will happen when everybody is asked about their point of view on a particular topic and everybody feels their point of view is taken seriously – even when it is different to other people attending the meeting.

It is very important that you don’t feel under pressure to make decisions you feel uncomfortable with during the process of making your PACT or include anything in your PACT document that you are not comfortable with.

As part of making your PACT you may find yourself thinking more about or talking about past situations which have felt very difficult and distressing – for example, about experiences of being admitted to hospital and given treatment against your will. You may wish to talk about how you might manage this distress with someone you trust in advance of your PACT meeting. If there are practical considerations which might make you feel more at ease it would be very helpful to be as direct as possible at telling health professionals what they could do to make you feel more comfortable.

When the PACT meeting is being arranged it might help to think about:

- Who you would like to attend: which health professionals, family members/friends, maybe an advocate or a peer supporter
- Where the meeting is going to be held: ideally a quiet room, with enough space for everybody to comfortably sit
- Whether you might need a table to sit round
- How long the meeting is going to last so everyone attending can make sure they have enough time
- Making sure you are free from other distractions e.g. not expecting any phone calls, arranging any necessary childcare
- A time of day when you feel you are at your best e.g. in the afternoon if you experience drowsiness from medication side effects in the morning
- What equipment you might need: a computer or pens and paper to make notes
- Who is going to lead the meeting: making sure it runs on time, making sure everybody gets to express their point of view and checking that you are not finding it too stressful

Filling in your PACT

Section 1: Personal details

This section is to help professionals find your records as quickly and easily as possible. This will be particularly helpful if you are being seen in a crisis outside your usual area or if you have moved around a lot. Try to include any alternative names you have used in the past.

It will probably be helpful to professionals working with you in a crisis to know something about your previous diagnoses and mental health history. This can include what others believe your mental health problems to be and your own opinions. However, if you have been living with mental health problems for a long time it might take a lot of time and space to go through it all. So, this section allows you to tell professionals where they can access that kind of information if they need it. For example, your electronic medical records, your GP or to ask a family member.

Section 2: Signs that I am becoming unwell

Health professionals who assess you when you are experiencing a crisis may not know what you experience when you start to become unwell. You can use this section to write down what you and the people who know you best understand to be the ‘early warning signs’ that a crisis is coming.

You might want to use some other tools to think through this. For example, you could use the Bipolar UK mood scale:

https://www.bipolaruk.org/Handlers/Download.ashx?IDMF=2898487b-0990-4b24-af7c-53946db9fc70

Some people have particular ‘code’ words they use with family or friends to talk about the early warning signs for example – things like ‘fizzy’, ‘hyper’ or ‘bouncy’.

Section 3: Key risks

This section is about thinking through the risks that are important to *you*. This may include thinking through things that have happened in the past that you want to avoid happening in the future.

There are lots of different types of risks that could be increased during a mental health crisis. Health professionals tend to think of three different main types:

- Risks to you
- Risks to other people
- Risks from other people

These risks include physical harm, emotional harm, problems in important relationships and harm to property or finances.

Section 4: Information for health professionals completing a mental capacity assessment

During a mental health crisis health professionals may carry out an assessment of that person's mental capacity to make particular decisions. Most commonly they assess whether a person is so unwell they have lost the capacity to make their own decision about taking particular medical treatments or being admitted to hospital. A PACT is designed to help professionals, who may not have met you before, carry out a ***personalised*** **mental capacity assessment**.

A health professional may go through the following checks according to a law called the Mental Capacity Act:

- Can the person understand all the information that is relevant to a particular decision,
- Can the person remember that information for long enough to help them make a particular decision
- Can the person use that information to help make a decision e.g. can they think through the pros and cons of that decision and apply it to their situation
- Can the person tell other people, in some way, the results of their decision

In the early stages, when someone is starting to become unwell, it can be difficult for health professionals to tell what is normal for that person. However, it is often the case that the person themselves or the family will know the signs that this person is becoming unwell and it is becoming difficult for them to make decisions in their usual way.

You can use this section to write down signs that you have lost the mental capacity to make decisions about mental health treatment and admission to hospital. These signs might fall into different categories:

- Changes in beliefs e.g. what you think about treatment options, unusual beliefs about having special powers or abilities
- Changes in behaviour e.g. becoming more irritable, overspending, being hyperactive
- Changes in speech e.g. speaking very fast, jumping from topic to topic

As an example, here is someone with bipolar talking about how their decisions around taking medication changes when they become unwell. They would like to use a document like a PACT to make sure they were offered a certain type of medication (Olanzapine) at an early stage in a crisis:

'I mentioned …taking Olanzapine and being sort of ok about that in a crisis. But I know that if I've gone beyond the sort of initial stages, I would refuse Olanzapine because …I open up that leaflet and it says one of the side effects… 'sudden unexplained death'….and it totally freaks me out, and I also think everyone doesn't have my best interests at heart'
Service User with bipolar

Section 5: Information for health professionals completing a Mental Health Act assessment

A Mental Health Act assessment will often involve an Approved Mental Health Professional (usually a specialist social worker), a doctor you do know and an independent doctor you may not have met before. This process is often referred to as 'being sectioned', as one outcome is being taken to hospital against your will which can be done according to a 'section' of the Mental Health Act. You can find more information about this process here: https://www.rcpsych.ac.uk/mental-health/treatments-and-wellbeing/being-sectioned

At the time a Mental Health Act assessment is done you may be very unwell and people can find the process confusing or distressing. You can use your PACT to tell professionals who may be assessing you in the future how professionals might know that an assessment is needed and how you would prefer the assessment to be done.

The Approved Mental Health Professional who carries out the Mental Health Act assessment has a legal duty to inform your 'nearest relative'. The law says how to work out who this is. Your PACT form has a section so you can say who you would like to be contacted in addition to your 'nearest relative' and you can also state reasons why you would prefer your official 'nearest relative' not to be contacted. The Approved Mental Health Professional may still have to contact your official 'nearest relative' but before doing so will consider carefully the reasons you have given.

You might want to think through

- When you need hospital admission: some people find that admission when they are in the earlier stages of becoming unwell helps them avoid some of the harms they have experienced in the past
- Where is the least stressful place to be assessed: this might be a particular setting like your home or you might find certain types of spaces easier to manage e.g. not small confined spaces

Section 6: Key crisis contacts

It is very important for you to have support during future mental health crises and you may be too unwell to contact people for yourself. The PACT form has a section to help health professionals contact the right people and ensure your privacy is maintained.

This could include requesting that your access to social media accounts is restricted to ensure you don't share information when unwell that you later regret.

Section 7: Confirmation of mental capacity to complete PACT

This section has a box for the health professional you are making your PACT with to confirm that you have fully discussed the PACT together and that they believe you have the mental capacity to complete this document. It is not necessary to have this assessment or for this box to be signed to complete this form. However, if you have a future mental health crisis and health professionals see confirmation that when you wrote your PACT form you had the

mental capacity to do so, they are likely to feel more confident in following your treatment refusals and requests.

**Tips for writing about treatment or care that you want/would prefer in Advance Statements:**<br>• Be **specific.** Try and write as much detail as possible so people assessing you in crisis have a clear picture of what you would prefer to happen<br>• Give **reasons.** Try and explain why you have made particular decisions so even if your preferences cannot be followed exactly health professionals can try and make sure the next best thing happens. For example, if there is a medication you would prefer to avoid if possible because of side effects but that you are willing to take in the short term for rapid improvement in your mental health.<br>• Be **realistic**. Mental health services may not be able to offer you everything that you would ideally like or would be most helpful in a mental health crisis. However, by writing down realistic preferences (e.g. treatment/care that you have had before which has been helpful) there is a higher chance some of your requests can be met.<br>• Be **prepared** that the clinician you make your PACT with may not agree with all your plans and preferences for a mental health crisis, they may not feel they can agree with all the sections of your PACT. It may be helpful for people using your PACT in a crisis to understand all the different points of view and why people disagreed about what might be best for you. Your PACT could be used to record the different opinions of people involved.

Section 8: Advance statement of wishes and feelings about community/Home Treatment Team care

The Advance statements are designed to be completed in the following order:
a) Completed by you before you meet with a health professional
b) Decisions/preferences which both you and the health professional aagrees should be followed in a crisis/during hospital admissions
c) Space for the health professional to write why they agree with the decisions you have made. If necessary the health professional could use this space to write down why they have a different opinion on some of your decisions or preferences.

You can use this section to help communicate your preferences and requests to a Home Treatment (or crisis) team who might see you if you become unwell in the future. You could put information here about ways the team could work with you to improve your mental health and/or you could refer back to the section on Mental Health Act assessment to help the community team judge when it is no longer possible for you to be treated at home without risking some of the harms you wish to avoid.

Some service users we have talked to wanted to use this section to give Home Treatment Teams ‘tips’ on how to persuade their future self to take medication in a crisis even if they said they didn't want to at the time.

You might find it useful to find out what it is possible for a crisis team to offer you to make sure you are clear on what sort of treatment options are available to you.

Section 9: Advance statement of wishes and feelings about the care I receive if I am admitted to hospital

If you need to be admitted to hospital, voluntarily or non-voluntarily the PACT form is designed to help you feel more involved in the decision making and assist the team on the inpatient ward feel more confident about what they can do to help your mental health improve as quickly as possible.

For many people who have been admitted to hospital and experienced treatment they did not agree to thinking about these experiences can be something they prefer to avoid. These experiences may have reminded them of traumatic events that happened even before they went into hospital. All this can make talking about and planning for possible future admissions very difficult. The health professional you are making your PACT with should offer you support and time to think about these experiences in a way that you find manageable. The hope would be to use the knowledge gained from these difficult experiences to avoid some of these things happening again in the future.

Sections 10 and 11: Advance statements of wishes and feelings about non-medical/medical treatments

You can use this section to make requests for medical and non-medical treatments. It is not possible to guarantee that you will receive these treatments but the health professionals you see should take your preferences into account and try to follow them where possible.

Section 12: Advance Decision to Refuse Treatments

You can use this section to make it clear you don't want particular medical treatments for your mental health problem (for example medications) even if you are too unwell to refuse them at the time. For example, if there are medications you have taken in the past that have caused bad side effects.

As long as you are not being treated under the Mental Health Act these advance decisions must be respected by health professionals. This is the case whether or not the clinician you are making your PACT with agrees with your decision.

If you are detained in hospital under the Mental Health Act, even though these advance decisions are no longer legally binding if they are about the treatment that you are getting for your mental health, any health professionals treating you should take them into account. If both you and the health professional you are making your PACT with agree with your advance decision health professionals can feel confident that this advance decision has been carefully thought about and is highly likely to be valid and applicable to you if you have a future mental health crisis. This may make it more likely that professionals will be happy to respect your advance decisions even when you are being treated under the Mental Health Act.

You can make an advance decision to refuse ECT which health professionals would have to accept even if you were detained under the Mental Health Act. However, if health professionals were extremely worried that your life would be in danger if you did not have ECT they could in some circumstances still give you this treatment.

PACTS are not designed for documenting advance decisions to refuse treatments which may be life-saving.

Section 13: PACT review plan

It is important you write down a plan for when you will review your PACT document. Some ideas for this could be:

- After any future mental health crisis
- After any future admission to hospital
- In 1 years time
- In 3 years time

If you do not manage to review your PACT within this time period it is still valid, but professionals who see you in future crises may feel less confident that your preferences and decisions apply to how you are in the present situation. They may then be less likely to follow your wishes.

Section 14: PACT access plan

It is important that people involved in your care are able to access your PACT in a crisis. Some ideas for storing your PACT are:

- Keep a paper copy at home
- Keep an electronic copy at home
- Give an electronic and a paper copy to a family member/friend
- Give a copy to your GP to upload on their records
- Give a copy to your mental health team to upload on their records
- Ask the police to place an alert on the Police National Computer system telling them that you have a PACT form
- Get a medic alert bracelet which says ‘advance decision’. Your PACT form can be stored electronically and accessed by health professionals at any time https://www.medicalert.org.uk/
- Get a ‘Lions’ bottle for your fridge which contains your PACT form. Emergency services know to look for the sticker alerting them to this when they enter someone’s home https://www.lions105d.org.uk/projects/miab.html
- If you have an iPhone use the ‘Medical ID’ notes section which can be accessed via the ‘emergency’ button on the lock screen

Section 15: Others involved in making this PACT

If other people you trust (e.g. family members/friends/care coordinator) have attended meetings to discuss your PACT and are involved in thinking about your care they can sign this section.

PACT summary page

This page is designed to be used by health professionals who might be working with you in urgent crisis situations when it is important to have information as quickly as possible.
It might be easiest to fill in this page after you have finished the more detailed sections.
