# Supplementary material for: The PACT advance decision-making template: preparing for Mental Health Act reforms with co-production, focus groups and consultation
Source: Int J Law Psychiatry. 2020 Jul-Aug;71:101563. doi: 10.1016/j.ijlp.2020.101563 (PMC7435693; doi:10.1016/j.ijlp.2020.101563)
Supplement: Supplementary file 3 — Health Professional PACT Guidance Notes Supplementary material 3 [file mmc3.pdf]

How to make and use a PACT

(Preferences and Advance decisions for Crisis and Treatment)

What is a PACT?

PACT stands for Preferences and Advance decisions for Crisis and Treatment. It is combines information that you might find in a mental health **crisis plans** with two documents that are described in the Mental Capacity Act 2005 (MCA): an **advance statement** and an **advance decision to refuse treatment**.

PACTs are focussed on improving service users experience of compulsory treatment and increasing their involvement in the medico-legal decision-making process. The PACT form contains sections to create a **personalised mental capacity assessment** and a **personalised Mental Health Act assessment.**

PACT forms and this guidance were developed following research with people who are living with a severe mental illness, family members of people severe mental illnesses, psychiatrists, AMHPs, lawyers and care coordinators.

Why should PACTs be made?

A PACT is a type of advance decision making (ADM) tool. There are ethical reasons for using these tools, policy recommendations and reasons revealed by the research that has been done on ADM in mental health contexts.

**Ethical Reasons**
ADM boosts the capability of service users to be involved in decision making about their own healthcare and treatment. It **maximises service user autonomy.**
Using ADM **lessens inequality** between those who are understood to have the capacity to make decisions about their healthcare and treatment and those who, at the time the decision is required, do not.
**Policy Recommendations**
NICE guidance on service user experience in mental health recommends developing advance decisions and advance statements with people who have severe mental illness particularly those who have previously been detained under the Mental Health Act 1983 (MHA) (CG136)
NICE guidance on managing Bipolar recommends encouraging people to develop advance statements (CG185)
**Evidence for ADM in mental health**
Surveys of people with mental illness consistently conclude that a majority are in favour of using ADM tools
When ADM tools have been created and analysed studies consistently find that service users typically write **clinically feasible** ADM documents.
Overall, the evidence suggests that ADM tools have the potential to **reduce the number of compulsory admissions**

Research on reasons people want to use ADM tools like PACTs suggests other reasons people give for wanting to use them are:

- The process of creating them can be therapeutic; providing peace of mind for the service user and greater involvement for families

'it can help families to come together around the illness'
(Family member of someone who has bipolar)

- They are empowering for the service user

'it empowers people to take a lot of responsibility for keeping themselves well and ….. their seeking out or accepting treatment when they're starting to relapse'
(AMHP)

- They facilitate communication between various health and social care teams

'GPs are very happy with this because it means that they can look and see what the psychiatrists want.'
(Service User)

Following the recent Independent Review of the Mental Health Act the government has accepted a recommendation to introduce statutory provision for 'advance choice documents'. This provision would offer people who are detained under the MHA rights to have advance written statements containing treatment preferences recognised and advance decisions to refuse treatment respected.

Making PACTs offers the opportunity to refine the process of creating these kinds of documents in anticipation of the coming legal changes.

Who is a PACT most useful for?

PACTs focus on improving the experience of compulsory treatment and particularly on enhancing the service users' ability to *request* compulsory treatment i.e. to inform clinical decision making on thresholds for initiating compulsory treatment. This is most likely to be appropriate for service users with the following experience:

- Previous experience of MHA assessment and detention in hospital
- Multiple previous crises
- Willing to engage with mental health teams when well

PACTs are *not* likely to be useful for service users who:

- Have no, or minimal (e.g. one episode only) experience of MHA assessment and detention in hospital
- Wish to refuse all treatments
- Do not experience fluctuating decision-making capacity

Those who wish to refuse all treatments may be better advised simply to make an Advance Decision to Refuse Treatment (but should be aware that it will not necessarily prevent treatment for mental disorder if they are detained under the Mental Health Act 1983).

Those who do not experience fluctuating capacity (i.e. those whose mental capacity to make decisions about treatment does not return between episodes of acute relapse of their mental illness) may be better suited to being as involved as possible in making care plans and crisis plans with their clinical team.

Are PACTs legally binding?

The following table outlines the types of document which are contained in a PACT and which sections of the PACT are legally enforceable (i.e. binding).

**Type of document**	**Relevant legislation**	**Is it legally binding?**
Advance statements of wishes and feelings	Mental Capacity Act 2005	No
Advance decision to refuse treatment (ADRT)	Mental Capacity Act 2005	Yes if the advance decision **exists**, is **valid** and **applicable** to the situation (for more detail on the meaning of these terms see pg. 7)<br><br>ADRTs about treatment of a mental disorder are not binding if the service user is detained under the Mental Health Act

Even if an advance statement or ADRT is not binding, it is good practice (and expected of them in both the Code of Practice to the MCA and the MHA) for health professionals to take them into account in decision-making. If an ADRT is not respected, the reasons should be clearly documented.

How should a PACT be made?

Making a PACT may involve several smaller meetings leading up to network meeting where all parties: service user, family member/friend/advocate and health professionals meet to finalise the content of the PACT. The flow chart below outlines one process which could be used to create a PACT. This process has been co-produced by service users, family members of service users and health professionals.

Interest in PACT

- Service user approaches health professional or health professional identifies PACT may be relevant for service user
- Health professional give service user PACT pack (electronic/paper template and guidance)
- Meeting arranged to create first draft of PACT
- Service user encouraged to discuss with family/friends/trusted others

↓

Drafting the PACT

- Meeting with one professional (could include family/friend)
- Look through PACT template
- Discuss unclear sections
- Draft content
- Arrange network meeting
- Encouraged to discuss further with family/friends/trusted others

↓

First draft sent to clinical team

↓

Making the PACT

- Meeting with service user, involved health professionals, psychiatrist/responsible clinician, family/friends/advocate
- Content discussed
- Confirmation of capacity to complete PACT documented
- Strategy for storage and access discussed
- Relevant document review date confirmed

↓

Cooling off period

- Final draft sent to clinical team for review and signing
- If concerns identified by any party further meeting arranged

Meetings to make a PACT

Research and consultation with service users, their family members and health professionals has identified the following factors as significant during meetings to draft documents like PACTs and proposed potential strategies to best manage these difficulties:

Difficulties	Strategies to manage difficulties
Potential for distress if recalling previous, difficult experiences of compulsory treatment	• Provide sufficient time for service user to take a break if required<br>• Supportive others available for service user<br>• Tissues available
Conflict and difficulties reaching group agreement on preferences	• It may be most helpful for those using the PACT in future to have a record of the conversations, which parties hold which opinions and their rationale.<br>• The health professional can choose not to endorse (sign) sections of the form which contain preferences that they do not believe would be helpful for the service user.
Undue influence: from clinical team or support network	• Explicit expression of this concern during the meeting<br>• Use of cooling off period
Managing power differentials: between health professionals, service users and members of social support network	• Offer service users choice wherever possible e.g. timing of meeting, venue<br>• Consider a neutral facilitator to chair the meeting<br>• Ensure all meeting participants encouraged to contribute<br>• Consider set up of the physical environment e.g. chairs in a circle<br>• Consider basic comforts e.g. drinks available, room temperature<br>• Document divergence in opinion<br>• Health professional or service user can choose not to complete or sign sections of the PACT that are impossible to come to mutual agreement on
Some service users may not have supportive social network	• Use of advocacy services/peer support
Writing a PACT may be challenging; how can the process be made as accessible as possible	• Consider time of the meeting e.g. afternoon when morning medication side effects may be reduced<br>• Use of 'conversation starter' prompts on PACT form<br>• Flexibility in process to draft PACT
Setting realistic expectations for clinical care in a crisis	• The legal limits of the PACT are outlined in service user and health professional guidance<br>• Part of the PACT discussion can involve exploring what would be realistic to expect from clinical services and what would be ideal but difficult to achieve

How is a PACT used in a crisis?

The following flow chart provides an example of how a PACT could be used to inform how a mental health crisis is managed.

Early signs of illness

↓

Contact with services

↓

PACT accessed

- From medical records
- From service user
- From family member/friends

↓

Other key crisis contacts informed by PACT

↓

If concerns about capacity to make decisions about care and treatment:
MCA assessment informed by PACT including

- Reference to personal indicators of loss of capacity to make decisions about treatment
- Reference to requests for Mental Health Act assessments i.e. thresholds for early assessment or alternative strategies

↓

If Mental Health Act assessment required:

- Reference to significant personal harm as defined in the PACT
- Reference to conditions of the assessment as outlined in the PACT

↓

Treatment plan informed by PACT

- Home/community treatment
- Inpatient treatment
- Medical and non-medical treatments

↓

Mental capacity assessments during course of treatment informed by PACT

- Indicators of return of capacity to make decisions about treatment and hospital admission

↓

Discharge planning informed by PACT

FAQs about using a PACT in a crisis

What if the health professional who assesses the service user in a crisis disagrees with the content of the PACT?

Only section 12 of the PACT: the Advance Decision to Refuse Treatments, is legally binding under the MCA and this is only the case (for treatments of mental disorder) as long as the service user is not detained under the MHA, for instance during an informal admission. There are also specific provisions in the MHA 1983 that relate to the status of advance decisions to refuse ECT and advance decisions to refuse medical treatment for mental disorder for patients on a CTO.

The assessing health professional may also have grounds to doubt that the ADRT **exists**, is **valid** and/or **is applicable** to the situation they are being asked to assess. The legal meaning of these terms is outlined below and can be found in full in section 25(2) of the Mental Capacity Act:

Legal term	Meaning/criteria
Exists	Was made by someone over the age of 18 who, at the time, had capacity to make it
Valid	Has not been withdrawn or contradicted by the person themselves or superseded by the person granting a power of attorney covering medical treatment
Applicable	Is applicable to the specific treatment and circumstances in question<br>N.B: PACTs are not designed to be applicable to decision making around life-sustaining treatment

In any of these cases, the health professional should seek legal advice as to whether it is possible not to follow the ADRT (and, if so, to document their rationale for doing so) or whether it is necessary to go to the Court of Protection to determine the existence, validity or applicability of the ADRT.

The preferences stated in other sections of the PACT function as advance statements. Although the health professional is under no statutory obligation to follow them it is good practice (as per the Codes of Practice for MCA and MHA) for the health professional to follow them as closely as possible and document rationale for deviating from these stated preferences as part of their crisis assessment. It is likely to be helpful for the service user to understand, in retrospect, why their preferences were not followed and to inform future PACTs.

What if an adverse outcome occurs after the recommendations in a PACT are followed?

PACTs facilitate the ability to set out contemporaneous evidence of capacity in relation to key matters, rather than having to rely after the event on retrospective reconstruction/normal practice so potentially reducing liability for clinicians involved in the drawing up of the PACT. There may be grounds for the actions of the treating clinician at the time to be questioned if an adverse outcome occurs when the recommendations in a

PACT are *not* followed (especially endorsed recommendations) and there is no documented rationale for deviation from the service user's advance decisions and preferences.

What if the resources the service user requests in their PACT are not available during a crisis?

It may be useful when making the PACT to distinguish between an ideal response to a crisis and what will be realistically available. This might include documenting alternative, less ideal scenarios. If the PACT is not following because appropriate resources are not available it would be good practice to document this. It is likely to be helpful for the service user to understand, in retrospect, why their preferences were not followed and to inform future PACTs.

What if the PACT is not found or not available when the crisis occurs?

As part of making a PACT a careful plan should be made about how to store the PACT so this scenario is avoided. Suggestions for this are outlined below.

A recent court case dealing with an end of life advance decision highlighted the role of GPs making hospital teams aware of any advance decisions that are contained on their records.[1] So, if the PACT is not found/available at the time of crisis as soon as anyone in the service user's network becomes aware that it may be relevant the treating team should be made aware of its existence and given a copy.

What if the PACT is out of date?

As part of making a PACT a review date or a set of circumstances should be decided by all those involved. If the PACT is out of date or the individual's circumstances are known to have changed dramatically since it was made it is not necessarily invalid. Health professionals assessing the individual in a crisis should not disregard the contents but the chances that the contents will be relevant to the situation in hand will be lower. It would be good practice for the health professional to document their rationale for considering the contents elapsed by time.

Filling in the PACT

Section 1: Personal details

This may be particularly important if the service user has a history of travelling out of area when unwell.

If there is a comprehensive summary of the service user's mental health history on their electronic records please specify the exact location, after consulting the service user. This section can be used to document the care team's view of relevant diagnoses and/or formulation and the service users view.

Section 2: Signs that I am becoming unwell

Resources to prompt discussions about crisis indicators include the Bipolar mood scale: https://www.bipolaruk.org/Handlers/Download.ashx?IDMF=2898487b-0990-4b24-af7c-53946db9fc70

[1] *NHS Cumbria CCG v Rushton* [2018] EWCOP 41

Section 3: Key risks

This section should include the service user and family member/friend's understanding of risk thresholds with a particular view to informing MHA assessments (see later section). Where disagreements exist about these thresholds this can also be documented.

Section 4: Information for health professionals completing a mental capacity assessment

The purpose of this section is to offer professionals assessing the service user in the future sufficient information to provide a confident, personalised assessment of the service users capacity to make decisions about healthcare and treatment.

The aim of the discussion around this topic is to paint a picture of how the service user might appear to an observer when they have lost the capacity to make decisions about healthcare and treatment.

As an example, here is someone with bipolar talking about how their decisions around taking medication changes when they become unwell. They would like to use a document like a PACT to make sure they were offered a certain type of medication (Olanzapine) at an early stage in a crisis:

'I mentioned …taking Olanzapine and being sort of ok about that in a crisis. But I know that if I've gone beyond the sort of initial stages, I would refuse Olanzapine because …I open up that leaflet and it says one of the side effects… 'sudden unexplained death'….and it totally freaks me out, and I also think everyone doesn't have my best interests at heart'
(Service User with Bipolar)

Section 5: Information for health professionals completing a Mental Health Act assessment

This section could be used to request a MHA assessment at a particular point in a person's pattern of relapse.

This information in this section can be used to inform MHA assessment, particularly if the individual has requested MHA assessment and detention at an early stage in a future episode of illness:

- **Nature** of the illness: For example, if a person is known to deteriorate very rapidly after early signs of relapse, community treatment has not been helpful in preventing this and a repeated pattern of risky harmful behaviour has been observed
- Detention in the **interests of the patient's own health:** This may include the psychological consequences of the harms that have occurred in the past when detention has been delayed
- Detention in the **interests of the patient's own safety:** This may include the service users own understanding of when their safety has been at risk
- Detention with a **view to the protection of other persons**:

This section can also be used to record preferences for how the assessment can be carried out in a way that involves family/friends (when preferred) and minimises distress

Section 6: Key crisis contacts

It may be important to probe how wiling the contacts listed on the PACT contact list are to be contacted in a crisis and what they might be able to realistically offer.

Section 7: Confirmation of mental capacity to complete PACT

This section requests that the health professional endorsing the PACT declares that the individual had the capacity to make it. Key issues to consider when completing this capacity assessment might be:

Understanding	• Understanding that a PACT is a document which aims to help plan for future mental health crises<br>• Understanding the legal and practical limits of a PACT: that treatment requests may not be practically possible or relevant to the particular circumstances of future crises<br>• Understanding risks and benefits of preferences stated in PACT e.g. risks and benefits of early compulsory hospital admission<br>• Ability to understand PACT information is not being severely impacted by distractibility or concentration difficulties
Retention	• Ability to retain this information
Use and weigh	• Ability to use and weigh their knowledge of PACTs and their knowledge of their own mental health history and consider whether PACTs will be helpful for them e.g. considering potential consequences of using a PACT vs not using a PACT<br>• Ability to use and weigh their knowledge of clinical services and treatments, including compulsory treatments and consider which options would be helpful for them e.g. comparing consequences of early admission compared with other options<br>• Ability to use or weigh information about the PACT is not being directly impacted upon by a delusion, or loss of awareness/appreciation, due to mental disorder<br>• Ability to use or weigh PACT information is not being significantly distorted, or made impossible, by extremes of negative or positive evaluation due to mood changes associated with mental disorder
Communication	• Ability to communicate these preferences. If they are not able to write the PACT themselves the ability to communicate, in any way, to somebody else who could write it for them.

Section 8: Advance statement of wishes and feelings about community/Home Treatment Team Care

The Advance statements are designed to be completed in the following order:
a) Completed by the service user prior to meeting with a health professional
b) Clear documentation of those decisions/preferences which the health professional also agrees should be followed in a crisis/during hospital admissions
c) Space for the health professional to further endorse shared decisions and give a rationale for why they do not support some of the service users decisions/preferences

There may be circumstances in which it is extremely difficult to discuss or endorse a particular service user's preferences. These scenarios could be considered as part of reflective practice. Options in these instances could include referral to an alternative health professional who may be better placed to assist the service user in completing the document or offering advice about the use of alternative documents e.g. a stand alone Advance Statement of the service users own design or a stand alone Advance Decision to Refuse Treatment.

A service user may be treated by a community or Home Treatment Team when they become unwell despite lacking the capacity to consent to the team's care plans on the basis that it is in their best interests. This section can be used to record the service user's preferences in this circumstance. This may include preferences around:

- How they would like to be persuaded to take medication if they are reluctant
- How assertive the team should be in visiting at home if they refuse
- Thresholds around when MHA assessment should be considered by the Home Treatment Team

Section 9: Advance statement of wishes and feelings about the care I receive if I am admitted to hospital

The aim of this section is to record how the use of coercive treatments might be avoided and agreed preferences for how difficult situations when coercive or intrusive treatment is required should be managed. Such scenarios include: compulsory medication (orally or by injection), 1:1 observations, restraint, seclusion. Recalling these experiences and explaining why previous experiences were traumatic may be particularly difficult for the service user. Sufficient time should be allowed to provide support.

Sections 10 and 11: Advance statements of wishes and feelings about medical and non-medical treatments

These sections can be used to make requests for medical for mental disorder (e.g. medication, ECT) and non-medical treatments (e.g. psychological therapies, OT, wellbeing practises). As part of this discussion it may be helpful to emphasise that such requests are not possible to guarantee and which requests are clinically realistic.

Section 12: Advance Decision to Refuse Treatments

This section makes explicit use of MCA provision for people to make advance decisions to refuse medical treatments for mental disorder e.g. medication, ECT. These decisions do not require the health professional to agree. However, the health professional can use the comments box to endorse refusals they do agree with or raise concerns about refusals they believe would be non-beneficial.

Legal impact of this Advance Decision to Refuse Treatment if the service user is receiving mental health community treatment or is admitted to hospital informally

An Advance Decision to Refuse Treatment does not need to be agreed with health professionals to be recognised under the Mental Capacity Act. It is legally binding for health professionals who are providing community treatment (e.g. Home Treatment Teams) or during an informal admission.

Legal impact of this Advance Decision to Refuse Treatment if the service user is admitted to hospital under the Mental Health Act 1983

An Advance Decision to Refuse Treatment for mental health problems is not legally binding if the person is admitted to hospital under the Mental Health Act 1983. However, according to the Mental Health Act Code of Practice it should be respected whenever possible[2]. It is expected that when a decision to refuse treatment has been discussed and shared with other health professionals this will add further *clinical* weight to the importance of this advance decision when making treatment plans in circumstances where the advance decision is not *legally* binding. If this decision is not respected the reasons should be clearly documented.

Special circumstances apply in the case of refusals of ECT (including advance refusals), which can be overridden if the conditions in s.62 MHA 1983 are met. Again, the same principles as set out immediately above apply in terms of considering the weight to be placed upon the advance decision in such circumstances.

PACTs are not designed for documenting advance decisions to refuse life-saving medical treatment, because they do not meet the statutory requirements in the MCA for the creation of such advance decisions.

Section 13: PACT review plan

The review plan can be tailored to the individual service user's preferences and/or pattern of illness. Suggestions include:

- After any future mental health crisis
- After any future admission to hospital
- In 1 years time
- In 3 years time

If the PACT is not reviewed within this time period it is still valid. However, professionals who see the service user in future crises may feel less confident that the advance statements and advance decisions to refuse treatment are valid and applicable (under the

[2] Department of Health and Social Care (2017) Mental Health Act 1983: Code of Practice, Section 24.2 and 24.6

MCA) to the crisis. Therefore, the preferences and decisions expressed in the PACT may be less likely to be followed.

Section 14: PACT access plan

The PACT storage plan can be tailored to the individual's situation. Ideas for service users to store their PACTs include:

- Keep a paper copy at home
- Keep an electronic copy at home
- Give an electronic and a paper copy to a family member/friend
- Give a copy to your GP to upload on their records
- Give a copy to your mental health team to upload on their records
- Ask the police to place an alert on the Police National Computer system telling them that you have a PACT form
- Get a medic alert bracelet which says 'advance decision'. Your PACT form can be stored electronically and accessed by health professionals at any time https://www.medicalert.org.uk/
- Get a 'Lions' bottle for your fridge which contains your PACT form. Emergency services know to look for the sticker alerting them to this when they enter someone's home https://www.lions105d.org.uk/projects/miab.html
- If you have an iPhone use the 'Medical ID' notes section which can be accessed via the 'emergency' button on the lock screen

Section 15: Others involved in making this PACT

Others who have attended key meetings and who wish to signal their involvement e.g. family members, should use this space to sign.

PACT summary page

This page is designed to be used by professionals seeing the service user in crisis e.g. Psychiatric Liaison Teams, Home Treatment Teams. It should be completed as a summary after the main document has been created.
